# Genome-wide SNP data unveils the globalization of domesticated pigs

**DOI:** 10.1186/s12711-017-0345-y

**Published:** 2017-09-21

**Authors:** Bin Yang, Leilei Cui, Miguel Perez-Enciso, Aleksei Traspov, Richard P. M. A. Crooijmans, Natalia Zinovieva, Lawrence B. Schook, Alan Archibald, Kesinee Gatphayak, Christophe Knorr, Alex Triantafyllidis, Panoraia Alexandri, Gono Semiadi, Olivier Hanotte, Deodália Dias, Peter Dovč, Pekka Uimari, Laura Iacolina, Massimo Scandura, Martien A. M. Groenen, Lusheng Huang, Hendrik-Jan Megens

**Affiliations:** 1 National Key Laboratory for Pig Genetic Improvement and Production Technology, Nanchang, China; 2 Animal Breeding and Genomics, Wageningen University, Wageningen, The Netherlands; 3 Centre for Research in Agricultural Genomics (CRAG), CSIC-IRTA-UAB-UB Consortium, Bellaterra, Barcelona Spain; 4 Institut Catala de Recerca i Estudis Avancats (ICREA), Carrer de Lluís Companys, Barcelona, Spain; 5 All-Russian Research Institute of Animal Husbandry named after Academy Member L.K. Ernst, Dubrovitzy, Moscow Region Russia; 6 Institute of Genomic Biology, University of Illinois, Urbana, Champaign, IL USA; 7 Division of Genetics and Genomics, The Roslin Institute, R(D)SVS, University of Edinburgh, Edinburgh, UK; 8 Animal and Aquatic Sciences, Chiang Mai University, Chiang Mai, Thailand; 9 Division of Biotechnology and Reproduction of Livestock, Department of Animal Sciences, Georg-August-University, Göttingen, Germany; 10 Department of Genetics, Development and Molecular Biology, Aristotle University of Thessaloníki, Thessaloniki, Greece; 11 Research Centre for Biology- Zoology Division, LIPI, Bogor, Indonesia; 12 School of Biology, University of Nottingham, Notttingham, UK; 13 Faculdade de Ciências and CESAM, Universidade de Lisboa, Lisbon, Portugal; 14 Department of Animal Science, Biotechnical Faculty, University of Ljubljana, Ljubljana, Slovenia; 15 Animal Breeding, Department of Agricultural Sciences, University of Helsinki, Helsinki, Finland; 16 Department of Chemistry and Bioscience, Aalborg University, Aalborg East, Denmark; 17 Department of Science for Nature and Environmental Resources, University of Sassari, Sassari, Italy

## Abstract

**Background:**

Pigs were domesticated independently in Eastern and Western Eurasia early during the agricultural revolution, and have since been transported and traded across the globe. Here, we present a worldwide survey on 60K genome-wide single nucleotide polymorphism (SNP) data for 2093 pigs, including 1839 domestic pigs representing 122 local and commercial breeds, 215 wild boars, and 39 out-group suids, from Asia, Europe, America, Oceania and Africa. The aim of this study was to infer global patterns in pig domestication and diversity related to demography, migration, and selection.

**Results:**

A deep phylogeographic division reflects the dichotomy between early domestication centers. In the core Eastern and Western domestication regions, Chinese pigs show differentiation between breeds due to geographic isolation, whereas this is less pronounced in European pigs. The inferred European origin of pigs in the Americas, Africa, and Australia reflects European expansion during the sixteenth to nineteenth centuries. Human-mediated introgression, which is due, in particular, to importing Chinese pigs into the UK during the eighteenth and nineteenth centuries, played an important role in the formation of modern pig breeds. Inbreeding levels vary markedly between populations, from almost no runs of homozygosity (ROH) in a number of Asian wild boar populations, to up to 20% of the genome covered by ROH in a number of Southern European breeds. Commercial populations show moderate ROH statistics. For domesticated pigs and wild boars in Asia and Europe, we identified highly differentiated loci that include candidate genes related to muscle and body development, central nervous system, reproduction, and energy balance, which are putatively under artificial selection.

**Conclusions:**

Key events related to domestication, dispersal, and mixing of pigs from different regions are reflected in the 60K SNP data, including the globalization that has recently become full circle since Chinese pig breeders in the past decades started selecting Western breeds to improve local Chinese pigs. Furthermore, signatures of ongoing and past selection, acting at different times and on different genetic backgrounds, enhance our insight in the mechanism of domestication and selection. The global diversity statistics presented here highlight concerns for maintaining agrodiversity, but also provide a necessary framework for directing genetic conservation.

**Electronic supplementary material:**

The online version of this article (doi:10.1186/s12711-017-0345-y) contains supplementary material, which is available to authorized users.

## Background

Domestication of pigs from wild boars occurred independently in Asia and Europe about 10,000 years ago [1]. Due to the biogeographic difference between wild ancestral populations, which results from 1.2 million years of separation, Asian and European pigs are genetically highly divergent [2–5]. *Sus scrofa* is native to Eurasia and North Africa, but was introduced into other parts of the world, i.e. into the Americas, primarily in its domesticated form, during the time of the European colonization in the sixteenth century, and later in Australia and New Zealand [6]. Both demographic processes, and natural as well as artificial selection, have led to the formation of a multitude of pig breeds around the world that vary in coat color, ear shape, body size, snout bluntness, behavior, growth rate, fatness, and prolificacy and other economically important traits.

In addition to domestication, crossbreeding between Asian and European indigenous pigs mediated by humans are significant landmarks in pig breeding history. Although anecdotal evidence exists even from the classical era, admixture between Western and Eastern pigs only started to become common in the mid- to late eighteenth century [7]. Introduction of Chinese pigs into Britain is documented from then and its aim was to improve the production characteristics of local pigs, which led to the creation of modern breeds such as Yorkshire (i.e., Large White), Berkshire and Hampshire [8]. In the late eighteenth century, Chinese pigs may also have been imported to America, and crossed with local pigs of European ancestry there [8], although most likely the Asian influence in American village pigs was through crosses with international breeds [9]. Reciprocally, at least since the 1840s, modern breeds such as Berkshire, Hampshire, Russian local pigs, Duroc, Large White and Landrace were introduced into China [10]. Such domestic animals were traded, loaded onto ships and released elsewhere. This is well documented, e.g., during the exploration of the Pacific by Captain Cook, who is credited for having released the first pigs on the New Zealand islands [11]. As in Europe, these imported pigs were used for crossbreeding with local breeds. However, in China, the introduction of pigs from outside and trading of pigs within China, appear to have been less widespread until recently, as is apparent from the high degree of geographic structure that remains in the Chinese traditional pig breeds [3]. Nevertheless, historical records and genetic evidence point to the contribution of European pigs to some East Asian breeds. For instance, the modern Korean Native pig is a cross between a local, traditional Korean pig and Berkshire. More recently, since the 1980s, Chinese pig breeders began programs to improve local breeds using Western stock [10] by creating synthetic breeds. In Africa, although advocated as an additional center of domestication, most of the evidence points to introgression from foreign breeds. Interestingly, Asian haplotypes predominate in East Africa, whereas European haplotypes predominate in West Africa [12].

Today, pig is a major livestock species, which in 2012 represented about 36.3% of the total meat production for human societies (www.fao.org), with major contributions from only a few international commercial breeds (i.e. Duroc, Large White, and Landrace). Nevertheless, hundreds of domesticated pig breeds worldwide [13] are still important for local meat production by small farmers. Many of these pig breeds have unique characteristics that differ from those of the international commercial breeds. Conservation of agrodiversity is one of the pillars to maintain food security, particularly in a rapidly changing world where consolidation of international plant and animal breeds is resulting in an increasingly narrow genetic basis for food production [14]. Thus, indigenous pig breeds, together with their wild relatives, are valued resources for the human society, not only for food, but also as genetic reservoirs. In addition, they constitute cultural and historical value since certain breeds are highly connected to local identity and specific agricultural practices. Finally, breed diversity can be leveraged for understanding the genetic basis of complex traits and adaptive evolution [15]. Large-scale genotyping technologies have enabled the analysis of the genetic ancestry and admixture of many domestic animals, including dogs [16], cattle [17], and pigs [18] and have also enabled the characterization of the genetic basis of phenotypic changes during domestication in chicken [19], dogs [20], rabbits [21] and pigs [22].

To date, population genetic studies using genomic data in pigs had a limited, usually regional, scope [9, 23–25]. Compared to previous generations of molecular markers, particularly microsatellites, single nucleotide polymorphism (SNP) markers allow for relatively straightforward data integration across studies since SNP genotypes can be compared unambiguously across studies. The aim of the current study was to perform a truly global integration of pig genotype data through the analysis of 1839 domestic pigs from 122 indigenous pig breeds that were collected in 29 different countries, together with 215 wild boars and 39 out-group individuals. As a result, our findings constitute a big leap in understanding the population structure, admixture, demographic history, and characterization of genetic loci involved in the domestication of pigs globally.

## Methods

### Samples and data

The raw Illumina 60K SNP data [26] of 3482 pigs, which include 3443 *Sus scrofa* and 39 non *Sus scrofa* suids (outgroups) (Table 1), were mainly obtained from three sources (see Additional file 1: Table S1): Wageningen University in The Netherlands (2464 individuals that encompass pig populations from Europe, Asia, Africa, Oceania, North America, international commercial pig populations, as well as outgroup suids), Jiangxi Agricultural University in China (821 individuals, which mainly consisted of pig populations from China, Russia and Ukraine), and the Autonomous University of Barcelona in Spain (197 individuals, which mainly represent pig populations from South America and Iberian pigs). Genomic SNP positions are based on the genome assembly Sscrofa 10.2 (EnsEMBL db version 83) [18].

**Table 1:** Number of populations and samples by continent

Subgroup	N_POPULATION_	N_SAMPLE_
Asian Domestic	40	624
Asian Wild	8	59
European Domestic	39	596
European Wild	10	149
American Domestic	19	222
American Feral	3	36
African Domestic	2	9
African Wild	1	7
Oceania Feral	1	10
Duroc	4	79
Landrace	7	129
Large White	4	76
Pietrain	3	58
Outgroup Suids	5	39

We conducted a series of quality control procedures on the raw data using PLINK v1.9 [27]. First, we excluded the breeds with less than five individuals. For the breeds or populations with more than 20 individuals, we randomly removed one individual from a pair of highly related animals (identity by state score > 0.95), and then kept the top 20 samples ranked by the SNP call rate. Next, we removed SNPs with a minor allele frequency (MAF) lower than 0.01, a call rate lower than 90% and individuals with a call rate lower than 90%, which resulted in a dataset of 55,072 SNPs for 2093 individuals that was used to estimate ROH, haplotype diversity and effective population size. We further removed SNPs with a MAF lower than 0.05, and in high linkage disequilibrium (r^2^ > 0.2) by using—maf 0.05 and—indep-pairwise 50 10 0.2 in PLINK v1.9 [27], respectively, and generated a dataset of 15,427 SNPs for 2093 individuals for subsequent multi-dimensional scaling (MDS), neighbor joining (NJ) tree and admixture analyses. See Additional file 2: Table S2 for further details.

### Statistical analysis

#### Population structure

MDS was carried out using—mds-plot and—cluster options in PLINK v1.9 [27] and visualized by R programming language [28]. The NJ tree was constructed using PHYLIP v3.69 [29] based on the identical by states matrix obtained by PLINK v1.9 [27], and visualized using FigTree v1.4 (http://beast.bio.ed.ac.uk/figtree). To facilitate visualization, we randomly selected six individuals from each population to build up the NJ tree. The geographical maps were plotted using R package MAPS [30] and MAPPLOTS (https://cran.r-project.org/web/packages/mapplots/). The coordinates of longitude and latitude of each population were set according to where the pigs were sampled (see Additional file 1: Table S1). The geographical distances between each pair of breeds were computed using *distm* function in R package GEOSPHERE (https://cran.r-project.org/web/packages/geosphere/). The proportion of mixed ancestry in the populations analyzed was evaluated by the ADMIXTURE 1.22 program [31]. We evaluated different K values with the mixed ancestry model (K = 2 to 17).

### Runs of homozygosity, haplotype diversity and effective population size

Runs of homozygosity (ROH) of each breed were identified using PLINK v.1.07 by a 5-Mb sliding window process across the genome with at least 50 SNPs, allowing five missing calls and one heterozygous SNP. The minimum length for ROH was set to 500 kb. ROH statistics were then transformed to F_ROH_. We inferred haplotypes of autosomes for all individuals using SHAPEIT v2 [32]. Haplotype diversity was calculated for populations with a minimum of 10 individuals. For each population, we randomly selected 10 individuals for the analysis. The haplotype diversity of a population was measured as the average number of haplotypes in windows of 5, 10, and 15 SNPs, respectively, which is similar to the method described in [16]. LD between adjacent SNPs was measured by the genotype correlation coefficient (r^2^) calculated by the—r2—ld-window 99999—ld-window-r2 0 command in PLINK v1.9. We used the same equation to fit the relationship between LD and genetic distance as previously described [33]. The SNPs used to calculate the LD within a population were filtered by applying the following criteria: a MAF higher than 0.05 and a *P* value for Hardy–Weinberg equilibrium higher than 1 × 10^−6^. The effective population size was estimated according to Sved [34], based on the equation r^2^ = 1/(4N_e_ *c* + 1), where r^2^ is the linkage disequilibrium between a pair of SNPs, N_e_ is the effective population size, *c* is the genetic distance in Morgan between a pair of SNPs, which was obtained by multiplying their physical distance and recombination rate [35]. The N_e_ at generation T, were obtained by the equation T = 1/2*c*, the same as described in [36].

### Domestication loci

To detect loci that may have been selected during domestication, we calculated the fixation index (*F*st) [37] between domestic and wild boars in Asia and Europe, separately. To avoid the influence of introgression, we used 42,808 SNPs (MAF > 0.05) in 782 individuals that have more than 90% of Asian or European ancestry in the analysis. We ranked the *F*st values of genome-wide SNPs, and genes within a 100-kb region of high-*F*st SNPs were identified as candidate genes that may have been involved in past selection. We selected candidate genes according to their functional relevance to phenotypes, such as e.g. behavior, development, energy metabolism, which may confer differences between domestic pigs and wild boars.

## Results

### Samples

After quality control, a dataset of 2093 samples representing 122 domestic pig breeds (1839 individuals), 19 wild boar populations (215 individuals) and five out-group populations (39 individuals) was available. The 122 domestic breeds included 104 local breeds or synthetic populations and 18 international commercial populations (Duroc, Large White, Landrace and Pietrain from different countries were considered as different breeds) (see Additional file 1: Table S1). Among the 104 local breeds, 39 originated from Europe, 40 from Asia, 22 from the Americas and three from other parts of the world. The wild boars were from widespread regions around the world and the five out-group populations are *Babyrousa babyrussa*, *Sus barbatus*, *Sus celebensis* and *Sus verrucosus* from the islands of Southeast Asia and *Phacochoerus africanus* from Africa. A more detailed description of many of these samples was reported in previous studies [9, 24, 25].

### Global population structure

**Fig. 1:**
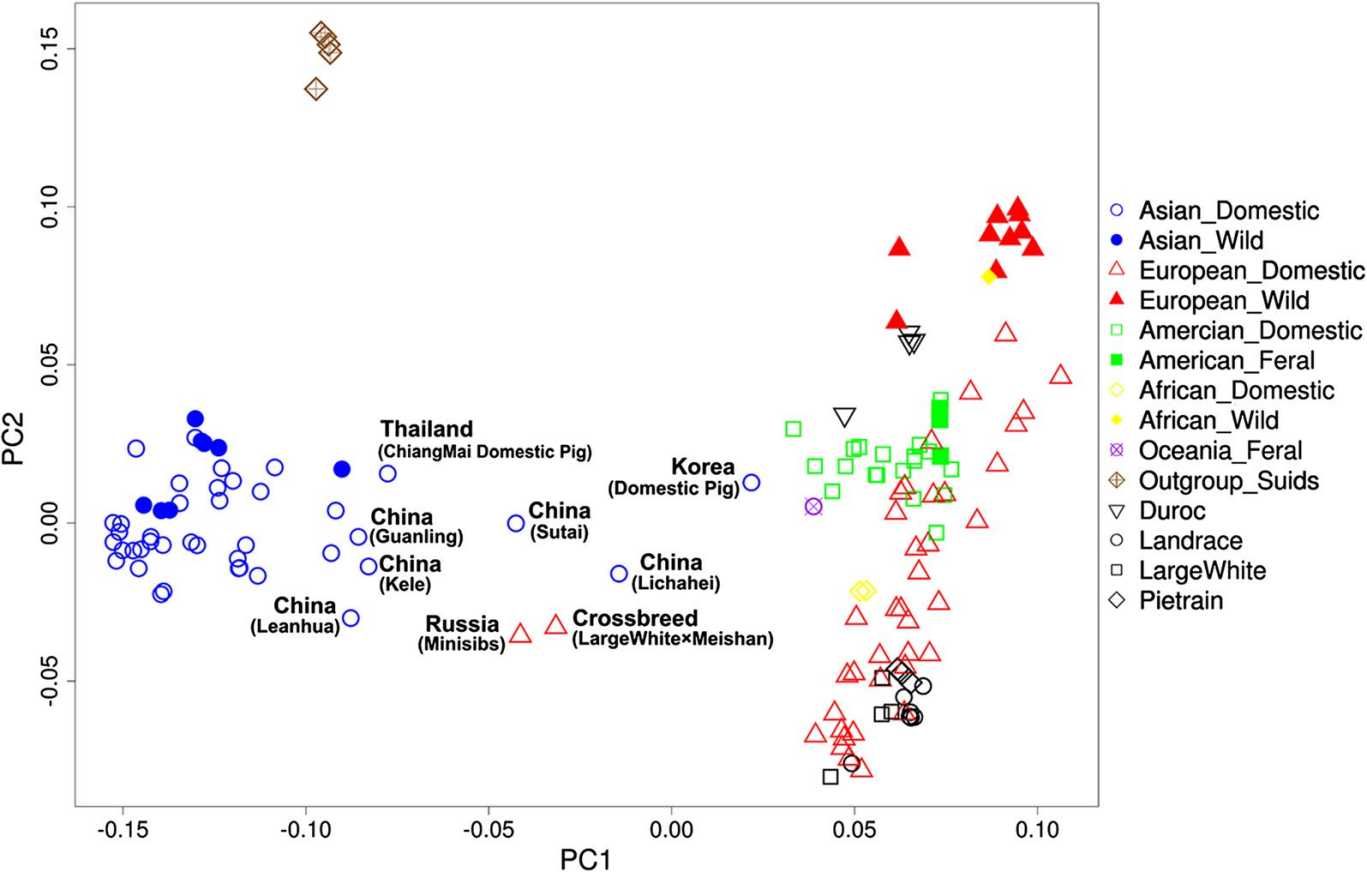
Global genetic structure of pig populations in this study. Multi-dimensional scaling analysis of pig populations from the five continents. Each point represents breed-average coordinates of eigenvalues of principle components 1 and 2. Points are mainly colored based on geographic origin of breeds; *blue* Asia, *red* Europe, *green* America, *yellow* Africa, *purple* Oceania, *brown* outgroup of Suids, *black* commercial Breeds; the pig populations in the middle of the *graph* representing admixed Asian and European ancestries are annotated

We performed multi-dimensional scaling (MDS) analysis on 15,427 SNPs for the 2093 individuals to investigate the genetic relationships between populations of domesticated pigs, wild boars and out-group populations. The first principal component separates Asian and European breeds (Fig. 1) and (see Additional file 3: Figure S1), in agreement with independent domestication and evolution of pigs in Asia and Europe. The American, African, and international commercial populations are much closer to European than to Asian pigs, which indicates a predominant contribution of European ancestry in the formation of these derived populations. Nevertheless, several populations are positioned in between the Asian and European main clusters. These include Oceania populations, populations from China (Lichahei and Sutai), the Korean native pig breed, Minisibs pigs from Russia and a Large White × Meishan F_1_ cross (Fig. 1) and (see Additional file 3: Figure S1). The intermediate position in the MDS reflects the fact that these populations are derived from both Asian and European pigs, and we validated the hybrid nature of these populations in subsequent admixture analysis. The Lichahei and Sutai pigs from China, the LW × Meishan F1 cross, and Minisib pigs from Russia consist of approximately 50% Asian and 50% European ancestries (see Additional file 4: Figure S2).Thus, the first MDS axis represents the Asian and European ancestries.

The second axis separates domestic pigs from wild boars in both Asia and Europe (Fig. 1) and (see Additional file 3: Figure S1), which indicates that domestication and subsequent artificial selection also resulted in the differentiation between wild and domesticated populations. Regarding the international commercial pig breeds, the Duroc breed tends to cluster with American domestic breeds and to separate from other commercial breeds (Landrace, Large White and Pietrain) that tend to cluster together. These results agree with the fact that Duroc pigs were originally developed in the USA, whereas, the other international commercial breeds (e.g. Large White-England, Landrace-Denmark, and Pietrain-Belgium) originated in Europe.

The results from hierarchical clustering (neighbor joining based on identity by state distance metric) generally agree with those from the MDS analysis. An important observation is that, even on a global scale, all populations of the major commercial breeds, still cluster together, i.e. breed identity has been maintained for both commercial and non-commercial populations (see Additional file 5: Figure S3).

### Regional population structures in Asia and Europe

**Fig. 2:**
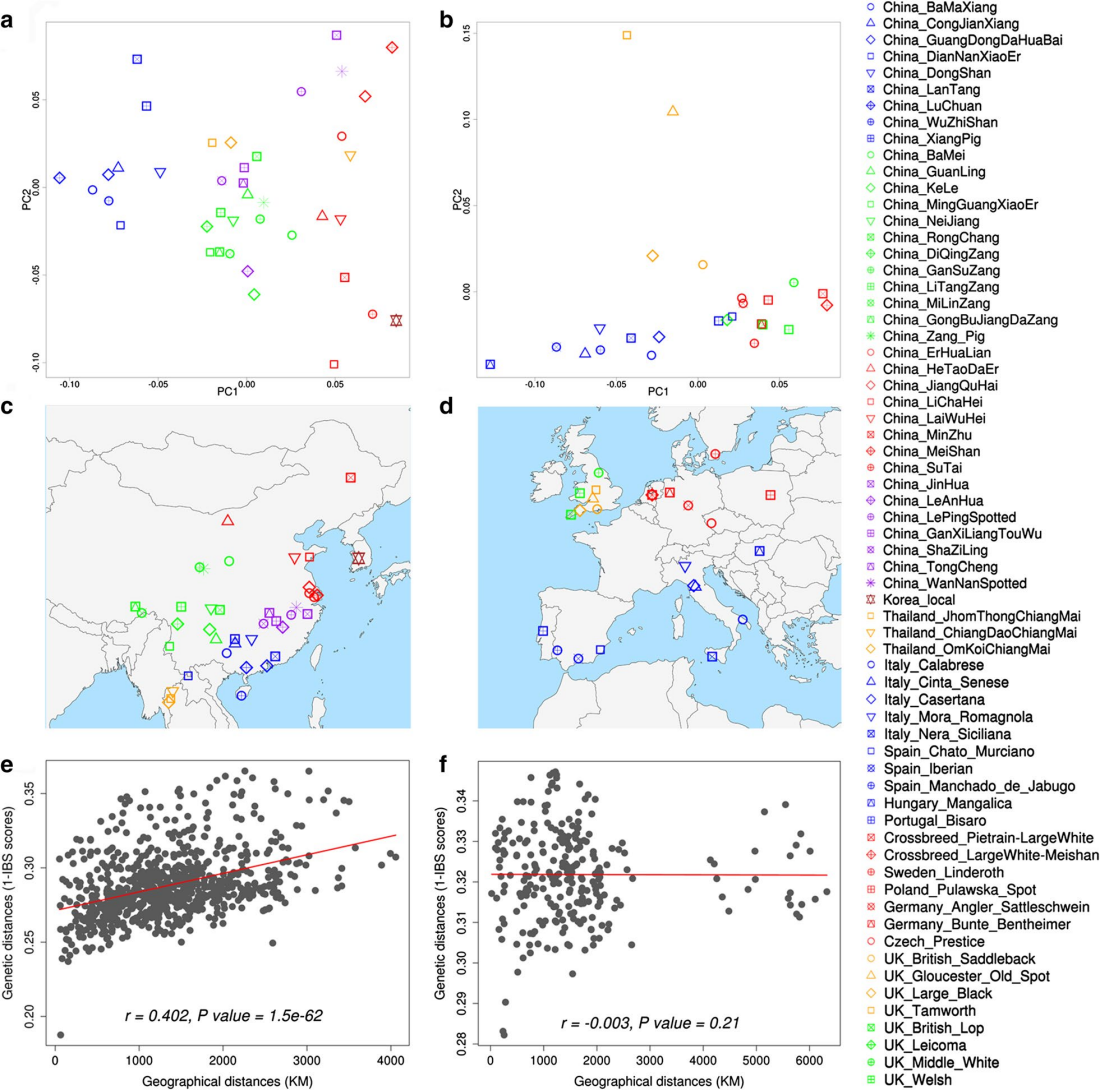
Regional genetic structure of indigenous pig populations in Asia and Europe. **a**, **d** show the results of the MDS analysis of pig populations from Asia (40 breeds) and Europe (26 breeds). Each *point* represents a breed, *colors* are assigned to each breed according to their geographical distributions, which are visualized in (**b**) and (**e**) for Asian and European pigs, respectively. **c**, **f** show the correlation between genetic and geographic distances among pig breeds in Asia and Europe, respectively. The legends of pig breeds are shown on the *right*. The upper legends in the *blue box* are for Asian breeds and the legends below in *red box* are for European breeds

To infer geographic region-specific details, we performed separate MDS analyses on pig populations within Asia and Europe, separately (Fig. 2). In Asia, most of the pig populations originate from China. In previous studies [3, 38], Chinese pigs were grouped into six categories that included pig types from Central China, the Yangtze River basin (East China), South China, Southwest China, North China, and Plateau (West and Northwest China), according to their external traits and geographical distributions [38]. The genetic clusters revealed in the MDS analyses are broadly concordant with this assignment into six categories (Fig. 2a, b). Pig breeds from South China and those from East and North China are located at each end of the first axis, while pig breeds from Central and West China are located in between (Fig. 2a, b). As expected under a model of gene flow between populations that is inversely proportional to physical distance, genetic distances were significantly correlated with geographical distances (Pearson correlation, *P* value = 1.5 × 10^−62^) (Fig. 2c). This is in sharp contrast to the results for pig breeds from the Americas where no concordance between genetic similarity and geographical distances was observed due to the complex colonization and breeding history of American pigs [9].

In Europe, the pig breeds from Southern Europe (Italy, Spain and Hungary) and those from North and middle Europe (Netherlands, Sweden, Poland, Germany and the Czech Republic) were genetically distinct. This distinction is represented by the first axis (Fig. 2d, e). Within the UK, British Lop, Leicoma, Middle White and Welsh breeds differ from Large Black, Gloucester Old Spot, Saddleback and Tamworth breeds. However, we observed no correlation with geographical distances among pig breeds in Europe (Fig. 2f), which is consistent with previous results based on microsatellites [3]. The absence of population structure in European pigs is explained, at least in part, by the Asian introgression and subsequent influence of highly productive “international” breeds on local pig diversity. In Europe, many ‘local’ or ‘traditional’ breeds have effectively become (partially) extinct due to such extensive crossbreeding [36, 39, 40].

### Global genetic ancestries

**Fig. 3:**
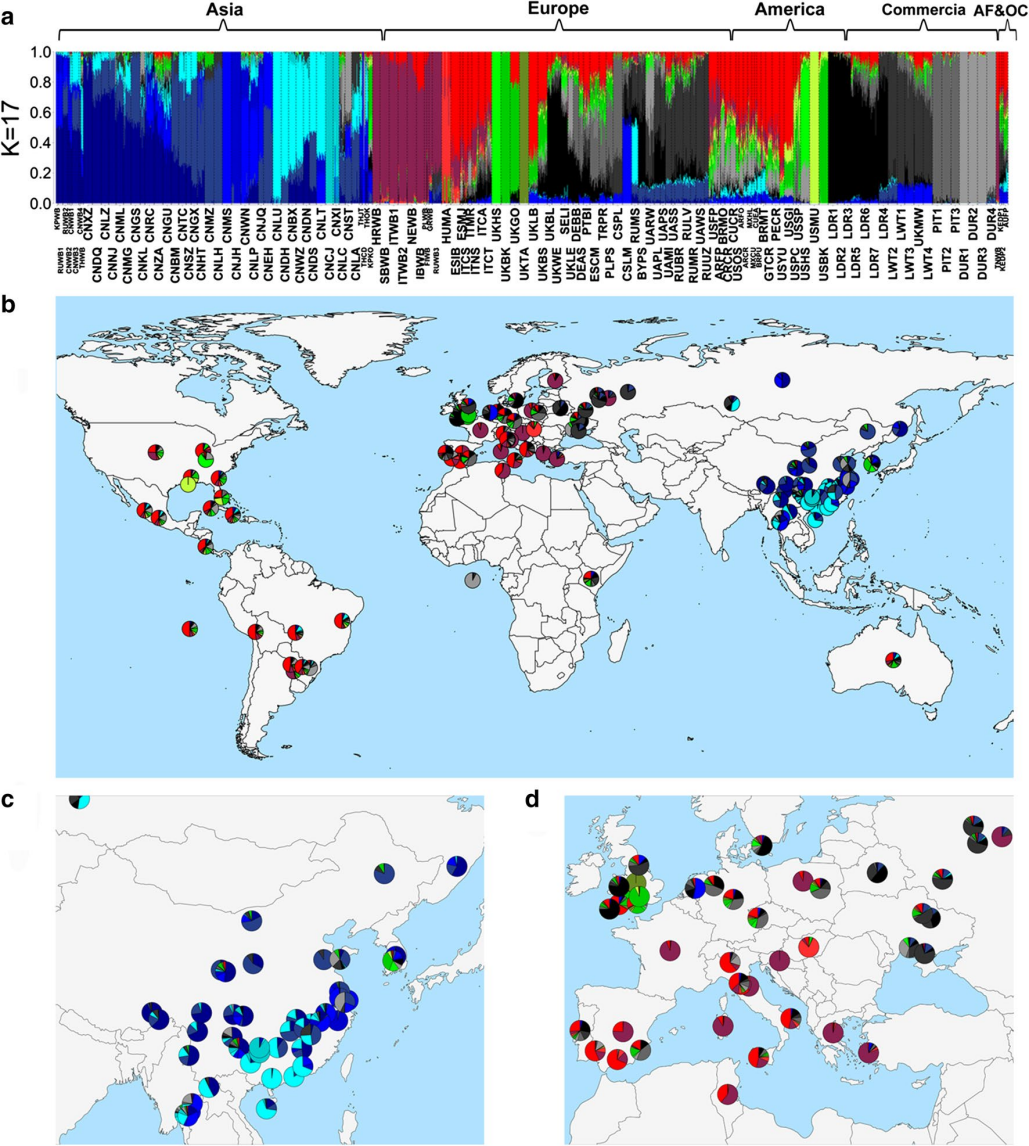
Landscape of worldwide admixture of pig populations. **a** Bar plot of admixture analysis (K = 17). Each *vertical bar* stands for an individual, the *colors* represent different ancestries. **b** Worldwide map of admixture for pig populations, the *pie plots* represent different breeds, the compositions of ancestries for a breed were calculated from the averages of ancestry composition of individuals within that breed. **c**, **d** Regional plot for Asian and European pigs, respectively

We further examined the genetic ancestry of pig populations worldwide by varying the number of ancestries (K) in ADMIXTURE v 1.2 [31] (Fig. 3) and (see Additional file 4: Figure S2). The population structure generally agreed with the MDS results. At K = 2, the two ancestries clearly reflect Asian and European origins. The pig populations from America, Africa, Russia and neighboring countries (including Ukraine, Belorussia and Kazakhstan) are mainly of European ancestry and (see Additional file 4: Figure S2). Even the international commercial pig breeds are mostly of European origin although they have a large Asian ancestry component [23]. At K = 8, we found two distinct Asian ancestries that are represented by pig breeds from East (Meishan, CNMS) and South (Luchuan, CNLU) China. The other six ancestries are represented by European wild boars, Hampshire (UKHS) and Berkshire (UKBK), and four international commercial breeds including Duroc, Large White, Landrace, and Pietrain and (see Additional file 4: Figure S2), (K = 8). At a higher K value (K = 17), we found various breed-specific ancestries that reflect a recent isolated breeding history (Gansu Zang (CNGS), Meishan (CNMS), Luchuan (CNLU), Jinhua (CNJH) and Congjiangxiang (CNCJ) pigs from China, Tamworth (UKTA), Hampshire (UKHS) and Berkshire (UKBK) from Europe, and Mulefoot pig (USMU) from USA).

### Admixture between Asian and European ancestries

An additional interesting pattern is the widespread admixed ancestries that we observed in pig populations across the different continents. Note that many breeds showed evidence of both Asian and European ancestries. Since these two lineages evolved independently, this admixture is a result of human-mediated activities. In Europe, importation of Chinese pigs is well accredited since the eighteenth century.

In Asia, 23 (57.5%) of the 40 breeds analyzed have 5% or more inferred European ancestry (see Additional file 4: Figure S2 and Additional file 6: Figure S4), (K = 2). Most of the European introgression was inferred to be from Duroc, Landrace, Large White, Berkshire and Hampshire breeds (see Additional file 4: Figure S2 and Additional file 6: Figure S4), (K = 13). Eight Asian breeds have more than 20% of genomic introgression from European pigs. These breeds include a Korean local breed (KPKO), a Thailand local breed (THCD), and six breeds from China (Lichahei (CNLC) from Shandong Province, Sutai (CNST) from Jiangsu Province, Kele (CNKL) and Guanling (CNGU) pigs from Guizhou Province, Leanhua (CNLAH) from Jiangxi Province, Minzhu (CNMZ) from Northeast China. Among these breeds with a large degree of European introgression (>20%), the Korean pig, a known East–West synthetic breed, formed the largest European ancestry group (see Additional file 4: Figure S2 and Additional file 6: Figure S4), (K = 2 and K = 13), including ancestry from Berkshire, Hampshire, Landrace and Duroc, which reflects the complex breeding history of this breed (Fig. 3a) and (see Additional file 4: Figure S2) (K = 13 and K = 17). This is largely in line with the known origin of the Korean local pigs. Sutai and Lichahei have been mainly admixed with Duroc, while Min pigs have a considerable contribution from Berkshire (Fig. 3a) and (see Additional file 4: Figure S2 and Additional file 6: Figure S4) (K = 8 and K = 17). It is interesting that the admixture with European pigs occurred mainly in Western and Northern Chinese pig breeds (Gongbujiangda (CNXZ) and Milin Tibetan (CNML) pigs, Kele and Guanling pigs from Guizhou Province, Mingguangxiaoer (CNMG) pigs from Yunnan province, Bamei (CNBM) pigs from Gansu province, Laiwuhei (CNLH) pigs from Shandong Province, Hetaodaer (CNHT) from Inner Mongolia and Min (CNMZ) pigs from Heilongjiang Province); the European ancestries that are involved encompass Large White, Landrace, Berkshire or other European breeds (Fig. 3a–c) and (see Additional file 4: Figure S2 and Additional file 6: Figure S4) (K = 13 and K = 17). In comparison, the pig breeds from South and Central China, including Erhualian, Xiang, Dongshan, Shaziling, Congjiangxiang, Lantang, Jinhua, Litang Tibetan and Luchuan, show no or negligible introgression from European pigs (Fig. 3a, c).

Iberian pigs from Spain, Cinta Senese and Nera Siciliana pigs from Italy, and Mangalica pigs from Hungary showed little evidence of influence from Asian pigs (see Additional file 4: Figure S2 and Additional file 7: Figure S5). By contrast, there is evidence of introgression from Asian pigs for all other pig breeds from Europe, including those from Ukraine and Russia (see Additional file 4: Figure S2 and Additional file 7: Figure S5). These results confirm the widespread Asian influences in European breeds.

The North and South American samples consisted mainly of village and feral pigs from eight countries [9]. Consistent with previous studies on pigs from the Americas using 60K SNP [9], and mitochondrial DNA data [41], pig populations from rural areas have mosaic genetic compositions that consist of multiple ancestries from both Europe and Asia. The largest ancestry components were similar to Iberian pigs (ESIB), in agreement with a primigenious origin from the Iberian Peninsula. Other European components are related to Duroc, Landrace, Berkshire, Hampshire, and European Wild boars (Fig. 3a) and (see Additional file 4: Figure S2 and Additional file 8: Figure S6). Intriguingly, a considerable contribution from pigs from both east and south China was observed in most of the American Village pigs (Additional file 8: Figure S6). In general, the village pigs from Brazil, Mexico and Cuba have larger Asian components than the pigs from other American countries (Fig. 3a).

In Africa, the Tunisian wild boar shows a high degree of similarity to the European wild boar, and specifically to the wild boar from the Iberian Peninsula. A local breed from Kenya was inferred to contain both Asian and European ancestries (Fig. 3a) and (see Additional file 4: Figure S2 and Additional file 8: Figure S6). This is in agreement with mtDNA studies, which showed that Asian haplotypes were abundant in East Africa but completely absent in the Northern African pigs (i.e. Tunisian wild boars) [41].

In Oceania, the Australian feral pigs also show admixed ancestry from Asian and European pigs (Fig. 3a) and (see Additional file 4: Figure S2 and Additional file 8: Figure S6).

The four major international commercial pig breeds, i.e. Duroc, Landrace, Large White, and Pietrain, have a considerable percentage of Asian ancestry (see Additional file 4: Figure S2 and Additional file 9: Figure S7) (K = 2). At K = 8, these four breeds form four distinct ancestries. The Landrace and Large White breeds also showed a diversity of genetic ancestries related to various pig populations including Berkshire, Hampshire, South European local pigs or Asian pigs.

### Genetic diversity

**Fig. 4:**
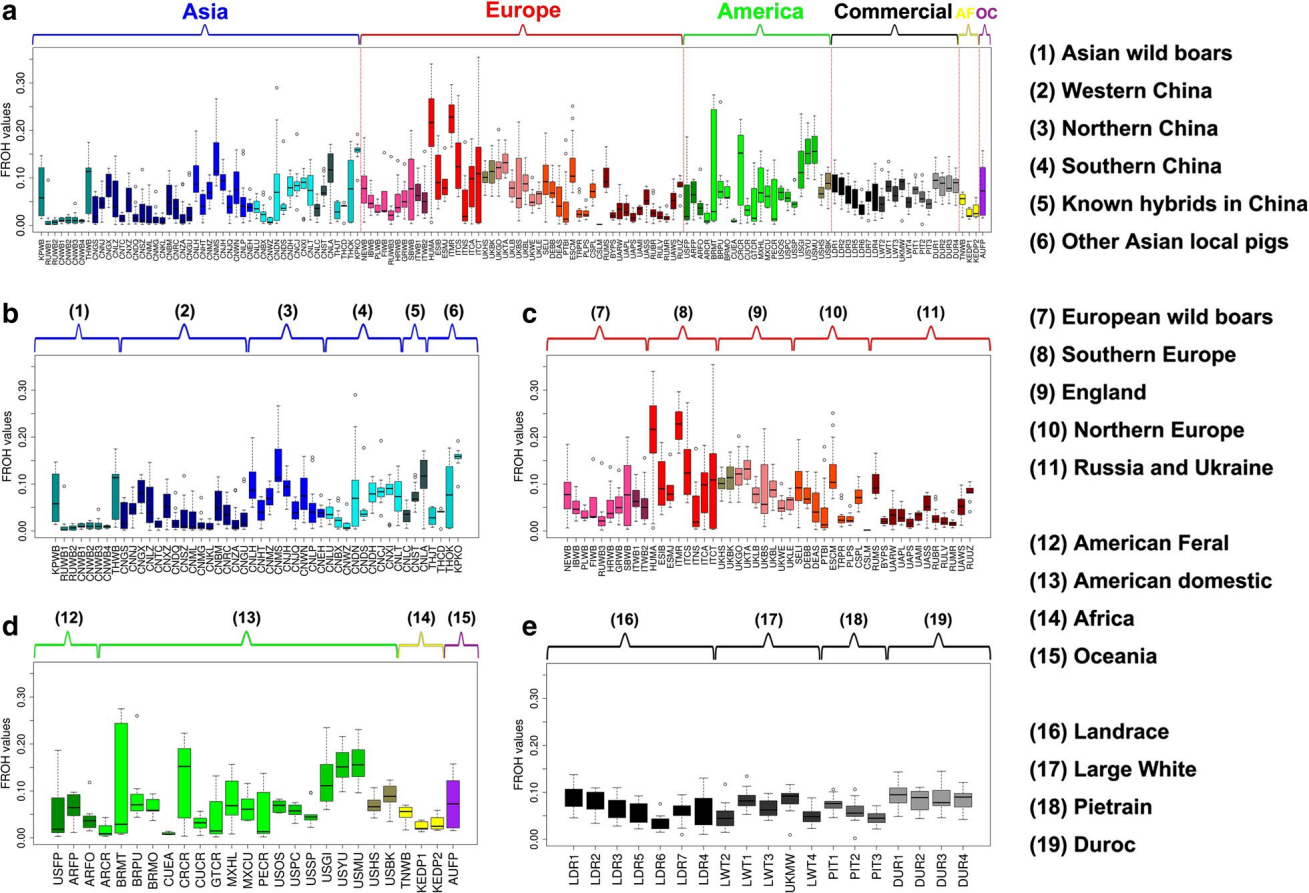
Distribution of F_ROH_ for pig populations across the world. **a** Box plot showing the global distribution of total length of F_ROH_ for pig populations worldwide, each point represents the total length of F_ROH_ for one individual, each *box* represents one breed, the *colors* and the order of breeds are the same as those described in Fig. 3. Regional view of F_ROH_ distribution for pig populations in Asia (**b**), Europe (**c**), America, Oceania and Africa (**d**), and international commercial breeds (**e**)

We analyzed runs of homozygosity (F_ROH_), haplotype diversity, and effective population size for each pig population to assess their inbreeding history and effective population size. Previous studies showed that 60K SNP data provide reasonably accurate estimates of long F_ROH_ [15]. We calculated the total length of ROH with a minimum length of 500 kbp for each individual. Considerable variation in F_ROH_ occurs within and across populations, which reflects the complex breeding history of pigs (Fig. 4). The cumulative length of ROH ranged from 4.98 Mb for the Dutch LW × Meishan F_1_ population from the Netherlands to 591.57 Mb for the Mora Romagnola pigs from Italy, and represented between 0.2 and 20.8% of the genome. Since the Dutch LW × Meishan is an F_1_ cross, it was expected to have few if any ROH. The 10 populations with the highest F_ROH_ included the Mora Romagnola (ITMR) and Cinta Senese pigs (ITCS) from Italy, the Mangalica breed from Hungary (HUMA), the Korea local breed (KPKO), the Mulefoot (USMU) and Yucatan Mini pigs (USYU) from USA, the Creole pigs (CRCR) from Costa Rica, the Gloucester old spot pigs (UKGO) and Tamworth pigs (UKTA) from UK, and the Leanhua pigs (CNLA) from China, which indicates that these populations have recently experienced considerable inbreeding (Fig. 4) and (see Additional file 1: Table S1). In addition, the total length of ROH was negatively correlated with haplotype diversity (Pearson correlation coefficient = -0.71, *P* value = 1.6 × 10^−20^) (see Additional file 10: Figure S8). Therefore, populations with a high F_ROH_ normally have a low haplotype diversity (Fig. 4) and (see Additional file 11: Figure S9). The effective population size (N_e_) for each population was estimated using linkage disequilibrium following the method described in [36] (see Additional file 1: Table S1). Considering only the populations with a minimum of 10 individuals, the estimated N_e_ of the past five generations is between 26 and 67. Even after accounting for a small systematic bias towards lower estimated Ne in populations that a have smaller sample size, it is clear that the N_e_ of indigenous pig populations is generally smaller than that of the international commercial breeds (see Additional file 12: Figure S10 and Additional file 1: Table S1).

### Loci involved in domestication

**Fig. 5:**
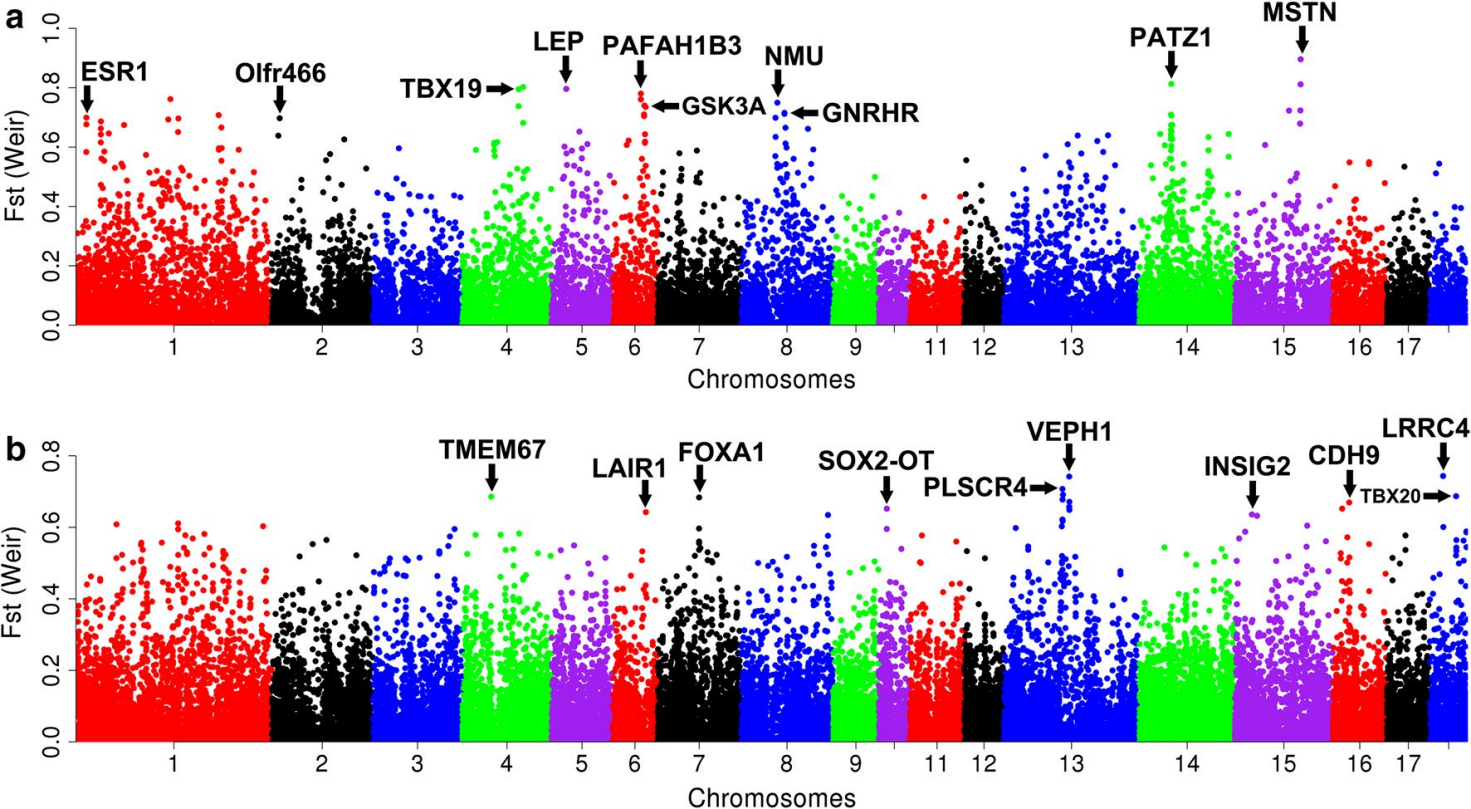
Genome-wide analysis of global *F*st between domestic pigs and wild boars. Manhattan plot of genome-wide *F*st values between domestic pigs and wild boars in Asia (**a**) and Europe (**b**). *F*st values are shown on the y axis, and genomic positions on the x axis. The different chromosomes are represented by different *colors*

Domestication and artificial selection have resulted in a wide range of phenotypes across domestic pig breeds that differ from their wild relatives. These are related to behavior, body size, fertility, locomotion ability and adaptation to feed provided by humans. To detect genetic loci that could be involved in the transition from wild to domestic, we calculated the genome-wide fixation index (*F*st) between domestic pigs and wild boars in Asia and Europe, separately (see “Methods” section) (Fig. 5). Empirically, we considered the 428 (1%) SNPs with the highest *F*st values as potential loci under recent (domestication) selection. Only six outlier SNPs were shared between Asia and Europe, which only slightly exceeds the number expected based on re-sampling of SNPs (see Additional file 13: Figure S11). Thus, we found no evidence for specific loci being under selection during the independent domestication processes in Asia and Europe. We examined the genes that are located within 100 kb to the top outlier SNPs with extreme *F*st values, and made the assumption that many of the genes around the top 30 outlier SNPs are involved in functions that are associated with phenotypic changes from wild to domestic (Fig. 5; Table 2). For Asian pigs, we identified genes related to muscle development (*MSTN* [42]), energy balance (*NMU* [43], *LEP* [44] and *GSK3A* [45]), social behavior (*TBX19* [46] and *PAFAH1B3* [47]), puberty and reproduction (*GNRHR* [48], *ESR1* [49] and *PATZ1* [50]) and perception of smell (*Olfr466* [51]) (Table 2). For European pigs, we identified genes related to growth and body development (*SOX2*-*OT* [52]), cardiac system development (*TBX20* [53]), metabolism of protein, glucose or fatty acid (*TMEM67* [54], *FOXA1* [55] and *INSIG2* [56]), central nervous system (*LRRC4* [57], *VEPH1* [58] and *CDH9* [59]), immune system (*LAIR1* [60]), and reproduction (*PLSCR4* [61]) (Table 2).

**Table 2:** Candidate genes for domestication loci in Asia and Europe

Region	Chr	Position	Fst	Rank	Location	Genes	Notes
Asia	15	105803885	0.90	1	Intergenic	*MSTN*	Muscle growth/differentiation [33]
	14	51279090	0.81	2	Intergenic	PATZ1	Spermatogenesis [41]
	5	23737420	0.80	6	In gene	LEP	Growth, energy homeostasis [35]
	4	90358483	0.80	7	In exon	*TBX19*	Personality traits angry/hostility [37]
	6	45563650	0.78	8	Intergenic	*PAFAH1B3*	Development of brain [38]
	6	45609490	0.76	11	In gene	*GSK3A*	Insulin signaling pathways [36]
	8	58493225	0.75	14	Intergenic	*NMU*	Food intake and energy balance [34]
	8	69912174	0.72	20	In exon	*GNRHR*	Pubertal delay [39]
	1	16779942	0.70	27	In gene	*ESR1*	Precocious puberty [40]
	2	12631241	0.70	29	In gene	*Olfr466*	Perception of smell [42]
Europe	18	21362149	0.74	1	Intergenic	*LRRC4*	Central nervous system [48]
	13	105334978	0.74	2	In gene	*VEPH1*	Central nervous system [49]
	13	94430998	0.71	3	In gene	*PLSCR4*	Reproduction [52]
	18	42219500	0.69	5	Intergenic	*TBX20*	Cardiac LDL-cholesterol [44]
	4	46284264	0.69	6	Intergenic	*TMEM67*	Protein catabolic process [45]
	7	67285122	0.68	7	Intergenic	*FOXA1*	Glucose homeostasis [46]
	10	12907670	0.65	15	In gene	*SOX2*-*OT*	Vertebrate development [43]
	16	15192108	0.65	16	Intergenic	*CDH9*	Autism spectrum disorder [50]
	6	53526888	0.64	18	Intergenic	*LAIR1*	Immune response [51]
	15	27308675	0.64	19	Intergenic	*INSIG2*	Cholesterol synthesis [47]

## Discussion

Pig is one of the most important livestock species for humans as a valued, global, resource for meat production and as an excellent animal model to understand the genetic mechanisms that underlie complex traits [6, 18]. Its long domestication history, originating from a large diversity of wild ancestors throughout Eurasia, and selection for economic and cultural purposes have resulted in a large number of breeds globally, which show a wide phenotypic diversity. Our worldwide survey on SNP data from 122 breeds/populations and 215 wild boars worldwide, reveals genetic ancestries, introgression and inbreeding histories of pigs at a global scale and at an unprecedented detail. Although there are potential issues regarding ascertainment bias [40] associated with the SNP assay used in this study, admixture analyses using 60K SNP and 30 million SNPs called from whole-genome sequence data provided very similar results (see Additional file 14: Figure S12), which indicates that robust conclusions can be drawn from the 60K SNP assay data for a wide population study as presented here.

### Population structure

The high degree of geographic structure observed here in the Asian domesticated pigs agrees with a previous report based on microsatellite markers [3], and differs substantially from that observed in pig populations from Europe and the Americas, in which almost no correlation between genetic and geographical distances exist [9]. The strong concordances between genetic and geographical distances for the pig populations in Asia may be attributed to the fact that pig populations within certain eco-geographical regions are more likely to have common ancestries, and that most of the breeds in Asia did not migrate over large distances. Furthermore, introgressions from European populations did not mask the identity of most Asian breeds, at least not to a large extent. Removing the breeds with more than 20% European introgression resulted in an increased correlation between genetic and geographical distances (see Additional file: 15 Figure S13). This indicates that admixture with geographically distant populations could be a major force in breaking regional genetic-geography concordance, as has been the case in Europe. Recent breed interchanges have largely masked an underlying geographic signal. However, it is interesting to note that some breeds have remained relatively unchanged for centuries. For instance, Ramirez et al. [62] showed that the modern Iberian breed is genetically very similar to a sixteenth century Spanish pig.

### Contribution of Chinese populations to worldwide pigs

The Chinese ancestries in European pigs observed in this study confirmed, on a broader population scale, the findings of previous genetic studies [2, 23, 41, 63]. These results are consistent with the historical record that South Chinese pigs were brought to England from Guangzhou in South China, the only treaty port city in China at that time, and have contributed to local British breeds, such as Berkshire and Yorkshire around 200 years ago [7, 8, 63]. Interestingly, our analyses revealed that ancestry represented by Lantang pigs from Guangdong Province is likely the major source of introgression in American pigs (Fig. 3a). The Meishan pigs of Eastern China, a breed famous for its high prolificacy, were imported to Western countries including France, England and USA in the 1980s [64]. Meishan pigs were used in experimental crosses to study the genetic basis of complex traits [65]. Recent studies showed that many Asian alleles with favorable phenotypic effects reached a high frequency in European pigs. These included *MC1R* alleles that are associated with black coat color [66], an *IGF2* allele for muscle growth [67], and *AHR* alleles for sow reproduction traits [23]. These studies underscored the importance of Asian pigs as vital genetic resources for international pig breeding and pork production.

### Contribution of European ancestry to worldwide pig populations

Both MDS and admixture analysis showed that European pigs were the major contributors to pig populations in those regions of the world where *Sus scrofa* does not occur natively (America, Africa, and Oceania). These results are consistent with mitochondrial and Y chromosome polymorphisms [41]. This contribution is due, in part, to the waves of colonization by Europeans since the sixteenth century. In addition, recent increase in worldwide trading of commercial, improved, pigs throughout the globe, and the desire of local farmers to improve their pigs using these western breeds, have likely contributed to this process as well. Populations in the Americas, Africa, and Oceania tend to harbor multiple ancestries of Mediterranean countries and/or international commercial breeds such as Berkshire, Hampshire, and Duroc (Fig. 3), which indicates a very dynamic process of global mixing of populations during several centuries.

More recently, the global process of mixing has become ‘full circle’ by the introduction of European pigs, themselves heavily influenced by Asian pigs, in Asia. In fact, one of the main original findings of our study is the widespread European influence in many Asian populations, the extent of which was mostly unknown until now. In Asia, we observed widespread and complex gene flow from European pigs, which indicates that many Asian indigenous pig breeds are no longer strictly Asian, but also contain a genetic component of European origin. Occurrence of European introgression in Japan [68], Korea [69] and Vietnam [70] was reported before. In China, there are over 80 pig breeds and a high diversity in phenotypes [64]. Historical documents indicate at least three waves of introgression from European pigs since the 1840s. The first wave of introgression may have occurred around the 1840s, when European pig breeds including Berkshire, Large White, Duroc, pigs from Russia, and Tamworth were brought to China by Germans and Japanese [10]. Subsequently, starting from the early twentieth century, probably since the 1910s, large-scale importation of Western European pigs, such as Berkshire in the Hebei, Sichuan, and Jiangsu provinces, and of Russian pigs in Northwest China, took place to improve local breeds. This study demonstrates that many pig breeds from West and North China contain ancestries from European pigs, notably Berkshire, Hampshire, Large White and Russian pigs, which is in agreement with historical records. Since 1937, war and civic and economic upheavals hampered systematic breeding, which may explain why most of the pig populations in China maintained their geographic identity in spite of admixture with European pigs. Since the 1980s, due to changes in Chinese policies regarding the introduction of foreign agricultural germplasms, many international commercial breeds were introduced into China, which gave rise to several synthetic breeds, such as the Lichahei from Shandong Province, and Sutai pigs from Jiangsu Province. Both breeds currently display considerable ancestry from Duroc.

### Indications for conservation of indigenous pigs

Inbreeding and decrease in effective population size may reduce the fitness of a population in response to challenges from changing environments or infectious diseases. This study provides an overview on the inbreeding, demography and admixture history of pig populations worldwide. First, our analysis revealed that 40 breeds or populations have substantial cumulative ROH (>200 Mb), and also exhibit low haplotype diversity, which indicate that these populations underwent recent inbreeding. This may reflect the fact that all domesticated and many wild populations are de facto under population management, deliberate or not. Second, we show that many of the indigenous pig breeds have smaller N_e_ than those of international commercial breeds. Since the commercial breeds are also the breeds that are the most admixed, this is not surprising. Finally, we found prevalent admixture of Asian and European ancestries in the indigenous pig populations, which suggests that many breeds have become less representative of the original local ancestries. The admixture of populations, particularly between East and West, has resulted in a re-shaping of the nucleotide diversity in the genomes of modern pig breeds. Because of that, only some of the current least admixed breeds may represent the original nucleotide and haplotype diversity in Europe. These results could help to make decisions on the conservation and management of pig populations. For example, Mangalica pigs from Hungary (HUMA) and Mora Romagnola pigs from Italy (ITMR) present the most extensive ROH in their genomes and the highest European ancestry, which indicate that these two breeds have undergone intensive inbreeding, and require special attention regarding conservation measures.

### Genetic basis of domestication

Domestication of plants and animals has been one of the major transitions in human history. Farming practices have not only altered the human societies but the interactions with nature, especially for domesticated plants and animals. Domestication of pigs has led to dramatic phenotypic changes transforming the wild boar into pigs by altering their behavior, morphology, coat color, reproduction and physiology. The admixture and MDS analyses presented in this study confirm the close relationship between wild and domesticated *Sus scrofa* in the geographic areas where domestication took place. Therefore, the genomic regions that show a much higher than average differentiation between wild and domesticated pigs should be enriched for loci under selection during domestication. We identified a number of genes that are located near loci with extreme *F*st values and that have functions that match the phenotypic changes from wild boars to domestic pigs. For instance, domestic pigs receive a stable feed supply from humans, while wild boars need to endure starvation if they cannot find food in the wild. We identified a number of genes with functions related to energy balance and metabolism (*NUM*, *LEP FOXA1* and *INSIG2*), which could have contributed to the adaptation of pigs to food scarcity or abundance. Genes involved in growth (*MSTN* and *SOX2*-*OT*) and reproduction (*GNRHR*, *PATZ1*, *ESR1*, and *PLSCR4*) could be associated with improved meat production and reproduction traits in domestic pigs that have undergone strong artificial selection. Lastly, genes related to nervous system and behavior (*TBX19*, *LRRC4*, *VEPH1* and *CDH9*) could be associated with changes in the behavior of domestic pigs compared to wild boars. The absence of signatures of selection found in previous studies [22–24] can be attributed to the higher density of SNPs, the specific selection-detection method, or the application of specific population contrasts in those studies. While further studies are needed to validate the role of the genes that we identified here in the domestication process, our findings confirm that this long-standing genetic experiment—i.e. domestication—is continuing to yield insights into biology and evolution.

## Conclusions

We present the largest population study on pigs and their wild ancestors to date, which investigates the population structure and introgression of worldwide pig populations globally. We demonstrate regional and global mixing of pig diversity, which reflect that this species has essentially followed many of the globalization events over the past centuries. Population diversity statistics such as ROH provided insight on inbreeding history and effective population sizes that allow us to recommend guidelines for breeding and conservation programs. Similar to other domesticated species, pigs represent an excellent model to study adaptation. We have identified a number of candidate genes that could have been under positive selection during domestication.

## Additional files

**Additional file 1: Table S1.** Detailed information and parameters of population genetics for pig populations in this study.

**Additional file 2: Table S2.** List of number of individuals and SNPs used in each step of analysis.

**Additional file 3: Figure S1.** MDS plot for all pig populations with detailed breed information.

**Additional file 4: Figure S2.** Neighbor-joining tree of pig populations under study.

**Additional file 5: Figure S3.** Population structure of each population revealed by the ADMIXTURE software at K = 2, 3, 5, 7, 10, 16. We marked the grouping of pig populations on the top of the graph to improve its readability. The first layer of the legend marks the five groups: Asia, Europe, America, Commercial and AF&OC (Africa and Oceania). The second layer of the legend further denotes more specific regions in corresponding continents: ASWB (Asian wild boars), CNDM_W (Chinese western domestic pigs), CNDM_N (Chinese northern domestic pigs), CNDM_S (Chinese southern domestic pigs), ① (Chinese hybrid pigs), ② (Southeast Asian pigs); EUWB (European wild boars), EUDM_S (European southern domestic pigs), UKDM (English domestic pigs), EUDM_N (European northern domestic pigs), RUDM (domestic pigs in Russia and its neighbor countries); AMFR (American feral pigs), AMDM (American domestic pigs); LD (Landrace), LW (Large White), PI (Pietrain), DU (Duroc); AF&OC (Pigs from Africa and Oceania).

**Additional file 6: Figure S4.** Expanded regional plot of Figure S2 showing the scenario of admixture for pig breeds and populations in Asia.

**Additional file 7: Figure S5.** Expanded regional plot of Figure S2 showing scenario of admixture for pig breeds and populations in Europe and Russia.

**Additional file 8: Figure S6.** Expanded regional plot of Figure S2 showing scenario of admixture for pig breeds and populations in North and South America.

**Additional file 9: Figure S7.** Expanded regional plot of Figure S2 showing scenario of admixture for pig breeds and populations in African and Oceanian countries.

**Additional file 10: Figure S8.** Scatterplot of the correlation between ROH length and haplotype diversity. Note that the total length of ROH and haplotype diversity are negatively correlated.

**Additional file 11: Figure S9.** Distribution of haplotype diversity for pig populations across the world. Diamonds and vertical bars represent means and standard deviations of number of haplotypes respectively in 5-SNP s (A), 10-SNP (B), and 15-SNP (C) windows across the genome for each population with a minimum of 10 individuals.

**Additional file 12: Figure S10.** Comparison of effective population size for domestic pigs and international commercial breeds.

**Additional file 13: Figure S11.** Observed number of shared SNPs between the top 1% SNPs with the highest *F*st values identified in Asia and Europe does not exceed the number of shared SNPs at random. The grey bars show the distribution of number of shared top SNPs at random, the red vertical line represents the observed number of shared top SNPs.

**Additional file 14: Figure S12.** Results of admixture analysis using 30.4 million SNPs called from whole-genome sequence data (A) were similar to those results obtained using 60K SNP data (B). Whole-genome sequence raw data from 188 individuals mainly obtained from [4, 18, 70], were used in the analysis. The whole-genome SNPs were called using GATK best practice workflow (www.broadinstitute.org/gatk). A total of 30.4 million SNP with a MAF >0.02 and a call rate >70% were kept for admixture analysis (A). A total of 44,988 SNPs with genome positions that were concordant with those of the Illumina 60K SNPs were extracted from the 30.4 million SNP data to represent the results of 60K SNPs (B).

**Additional file 15: Figure S13.** Scatter plot of geographical distances among pig breeds in China against their genetic distances after removing pigs breeds with more than 20% introgression from European ancestry as revealed by admixture analysis (K = 2).
