# Correction to: Genome-wide SNP data unveils the globalization of domesticated pigs

**DOI:** 10.1186/s12711-020-00549-3

**Published:** 2020-06-04

**Authors:** Bin Yang, Leilei Cui, Miguel Perez-Enciso, Aleksei Traspov, Richard P. M. A. Crooijmans, Natalia Zinovieva, Lawrence B. Schook, Alan Archibald, Kesinee Gatphayak, Christophe Knorr, Alex Triantafyllidis, Panoraia Alexandri, Gono Semiadi, Olivier Hanotte, Deodália Dias, Peter Dovč, Pekka Uimari, Laura Iacolina, Massimo Scandura, Martien A. M. Groenen, Lusheng Huang, Hendrik-Jan Megens

**Affiliations:** 1 National Key Laboratory for Pig Genetic Improvement and Production Technology, Jiangxi Agricultural University, Nanchang, China; 2 Animal Breeding and Genomics, Wageningen University, Wageningen, The Netherlands; 3 Centre for Research in Agricultural Genomics (CRAG), CSIC-IRTA-UAB-UB Consortium, Bellaterra, Barcelona, Spain; 4 Institut Catala de Recerca i Estudis Avancats (ICREA), Carrer de Lluís Companys, Barcelona, Spain; 5 All-Russian Research Institute of Animal Husbandry named after Academy Member L.K. Ernst, Dubrovitzy, Moscow Region Russia; 6 Institute of Genomic Biology, University of Illinois, Urbana, Champaign, IL USA; 7 Division of Genetics and Genomics, The Roslin Institute, R(D)SVS, University of Edinburgh, Edinburgh, UK; 8 Animal and Aquatic Sciences, Chiang Mai University, Chiang Mai, Thailand; 9 Division of Biotechnology and Reproduction of Livestock, Department of Animal Sciences, Georg-August-University, Göttingen, Germany; 10 Department of Genetics, Development and Molecular Biology, Aristotle University of Thessaloníki, Thessaloniki, Greece; 11 Research Centre for Biology-Zoology Division, LIPI, Bogor, Indonesia; 12 School of Biology, University of Nottingham, Notttingham, UK; 13 Faculdade de Ciências and CESAM, Universidade de Lisboa, Lisbon, Portugal; 14 Department of Animal Science, Biotechnical Faculty, University of Ljubljana, Ljubljana, Slovenia; 15 Animal Breeding, Department of Agricultural Sciences, University of Helsinki, Helsinki, Finland; 16 Department of Chemistry and Bioscience, Aalborg University, Aalborg East, Denmark; 17 Department of Science for Nature and Environmental Resources, University of Sassari, Sassari, Italy

## Correction to: Genet Sel Evol (2017) 49:71 10.1186/s12711-017-0345-y

After publication of this work [1], we noticed that information regarding the university is missing in the affiliation for Bin Yang, Leilei Cui and Lusheng Huang (affiliation 1).

The correct affiliation should be: National Key Laboratory for Pig Genetic Improvement and Production Technology, Jiangxi Agricultural University, Nanchang, China.
