# Supplementary figures and images for: Genome-wide SNP data unveils the globalization of domesticated pigs
Source: Genet Sel Evol. 2017 Sep 21;49:71. doi: 10.1186/s12711-017-0345-y (PMC5609043; doi:10.1186/s12711-017-0345-y)

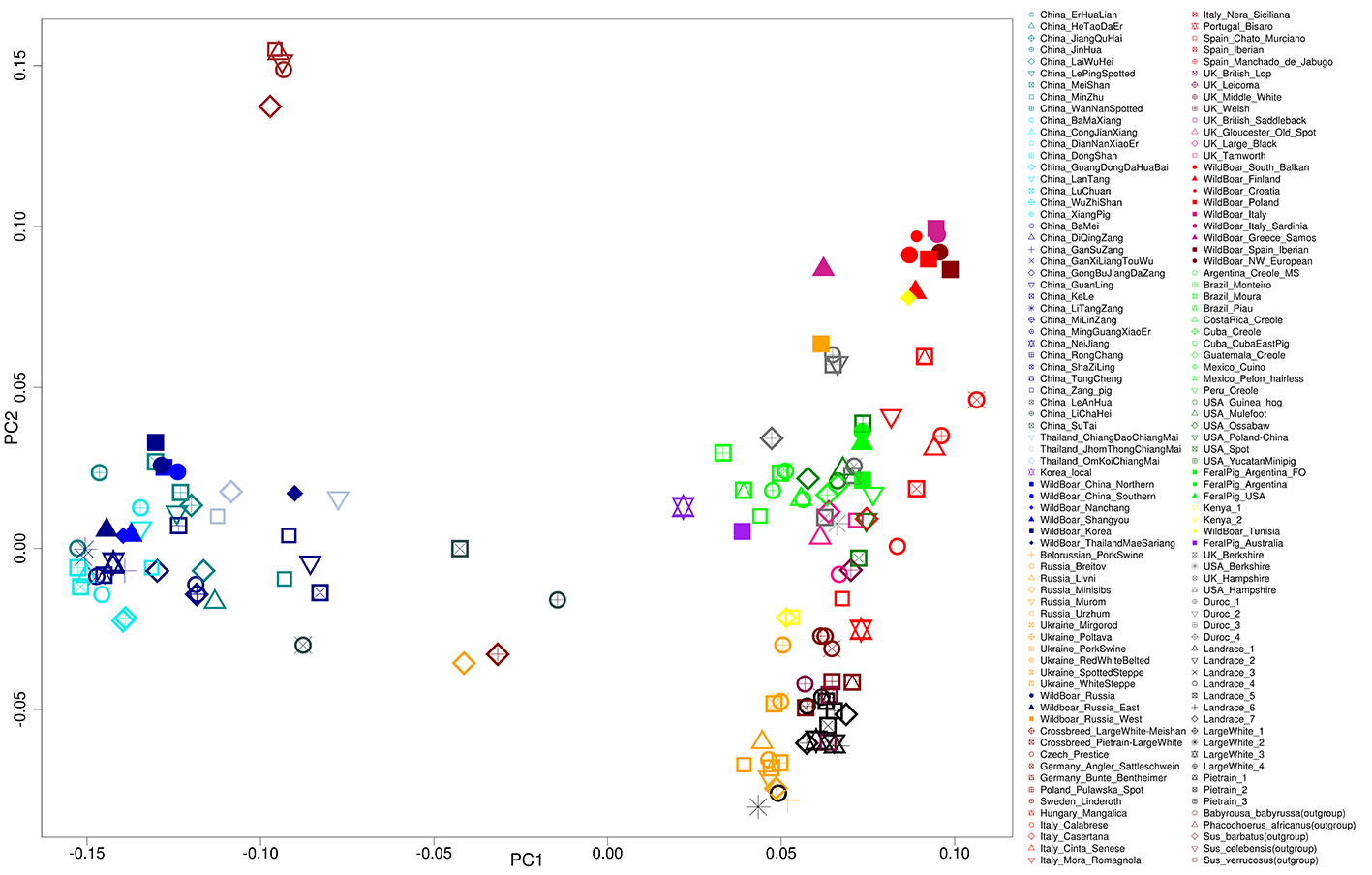

Supplement: Supplementary file 3 — Additional file 3: Figure S1. MDS plot for all pig populations with detailed breed information. [file 12711_2017_345_MOESM3_ESM.tif]

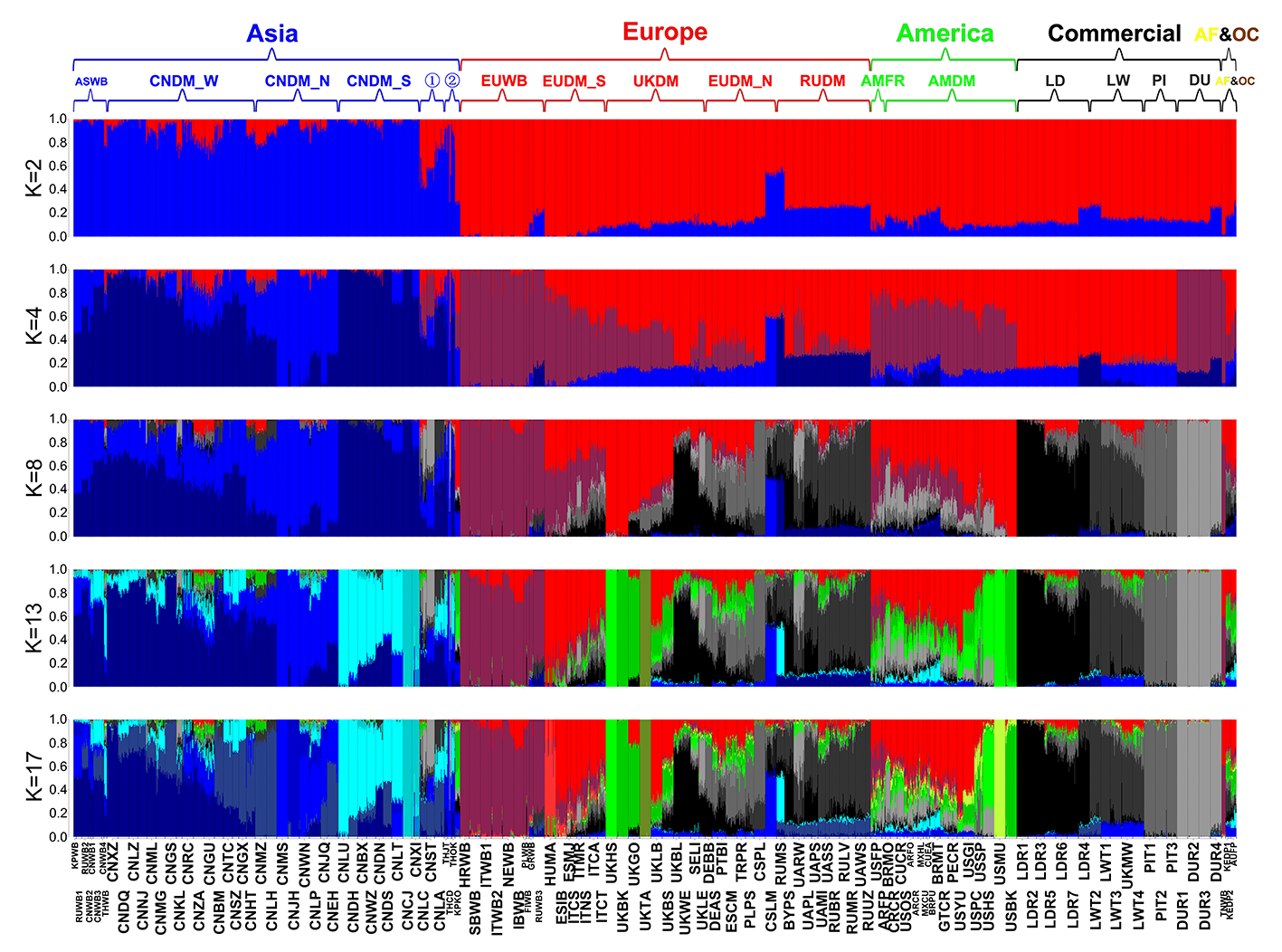

Supplement: Supplementary file 4 — Additional file 4: Figure S2. Neighbor-joining tree of pig populations under study. [file 12711_2017_345_MOESM4_ESM.tif]

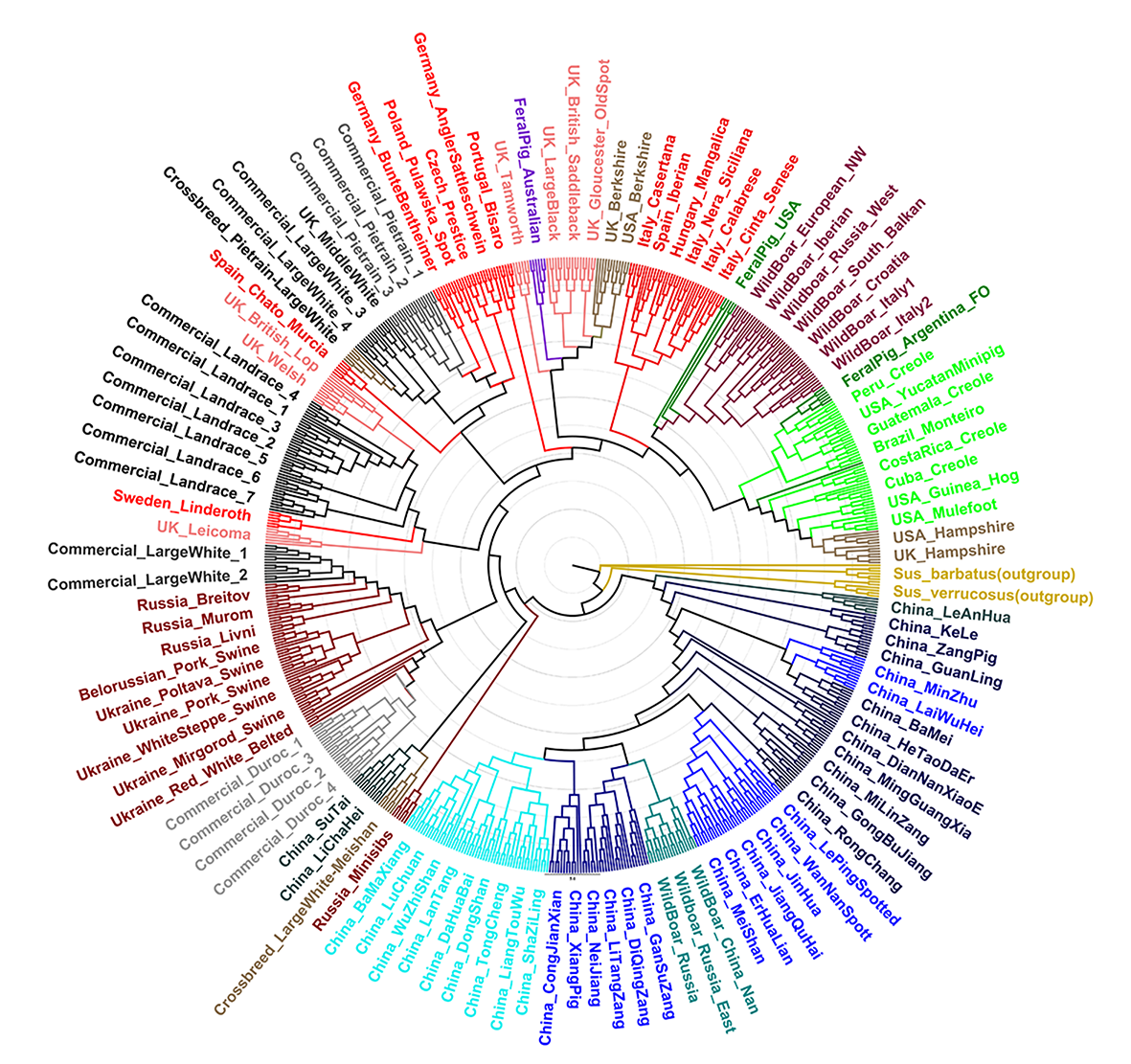

Supplement: Supplementary file 5 — Additional file 5: Figure S3. Population structure of each population revealed by the ADMIXTURE software at K = 2, 3, 5, 7, 10, 16. We marked the grouping of pig populations on the top of the graph to improve its readability. The first layer of the legend marks the five groups: Asia, Europe, America, Commercial and AF&OC (Africa and Oceania). The second layer of the legend further denotes more specific regions in corresponding continents: ASWB (Asian wild boars), CNDM_W (Chinese western domestic pigs), CNDM_N (Chinese northern domestic pigs), CNDM_S (Chinese southern domestic pigs), ① (Chinese hybrid pigs), ② (Southeast Asian pigs); EUWB (European wild boars), EUDM_S (European southern domestic pigs), UKDM (English domestic pigs), EUDM_N (European northern domestic pigs), RUDM (domestic pigs in Russia and its neighbor countries); AMFR (American feral pigs), AMDM (American domestic pigs); LD (Landrace), LW (Large White), PI (Pietrain), DU (Duroc); AF&OC (Pigs from Africa and Oceania). [file 12711_2017_345_MOESM5_ESM.tif]

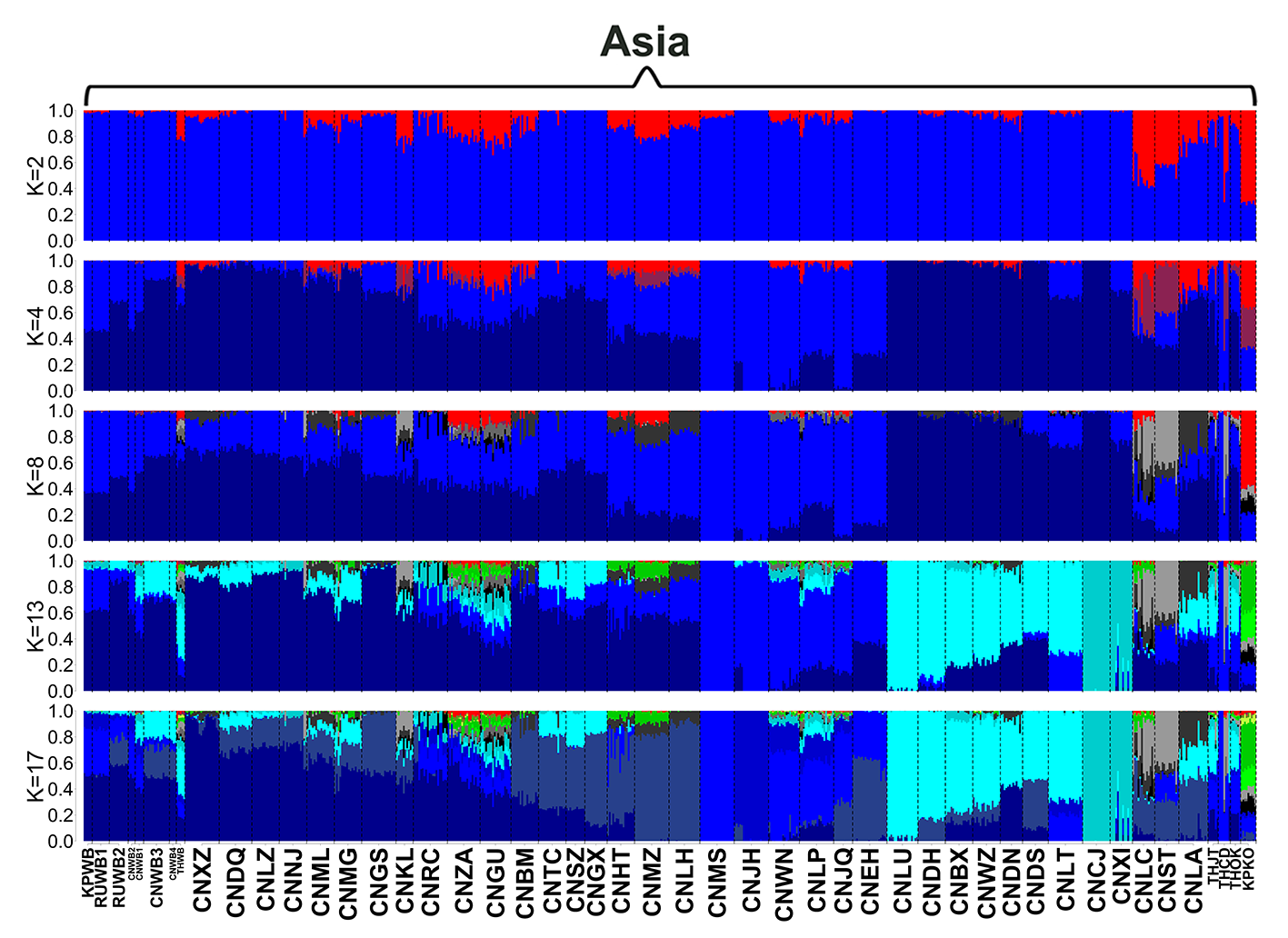

Supplement: Supplementary file 6 — Additional file 6: Figure S4. Expanded regional plot of Figure S2 showing the scenario of admixture for pig breeds and populations in Asia. [file 12711_2017_345_MOESM6_ESM.tif]

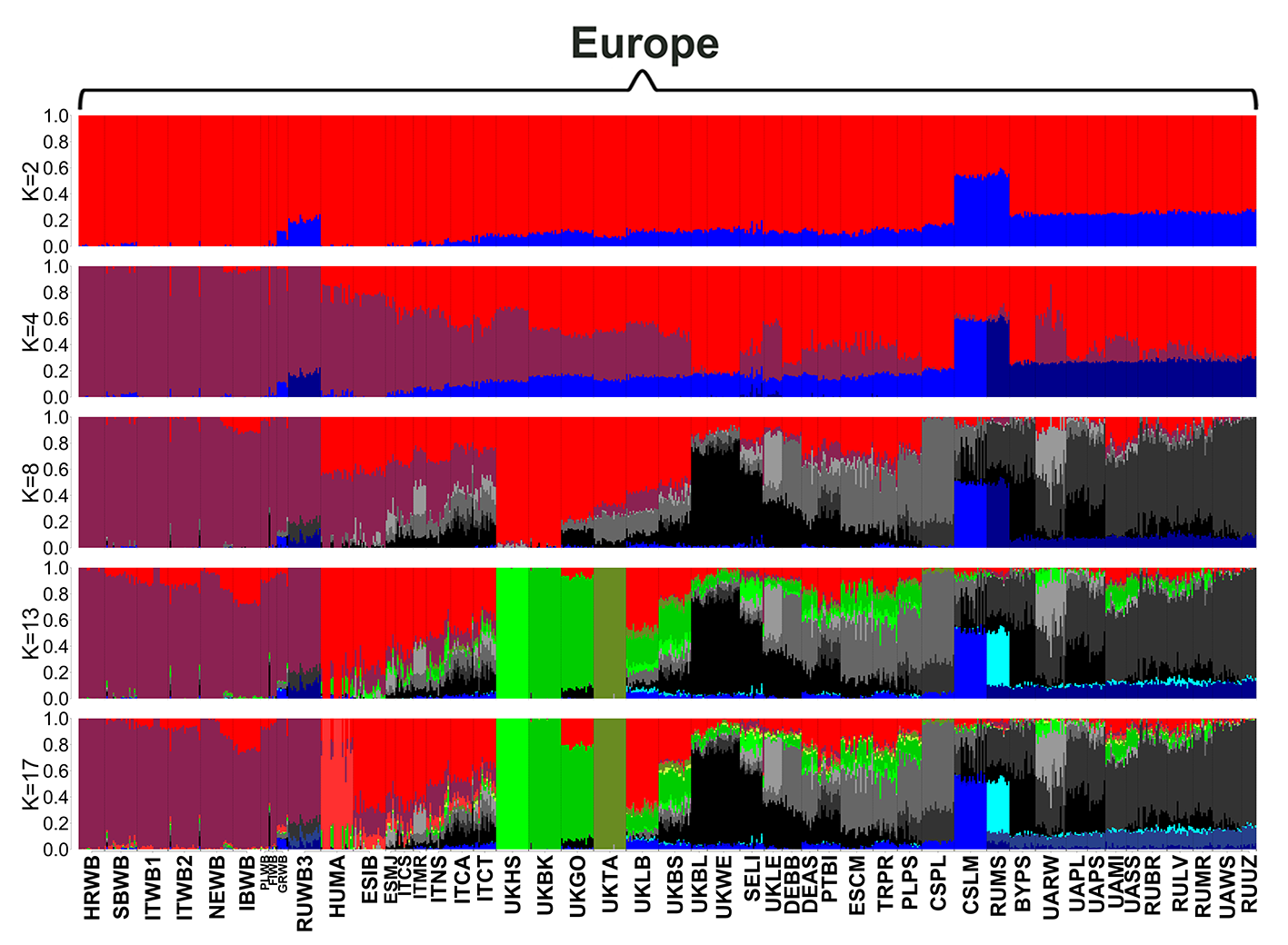

Supplement: Supplementary file 7 — Additional file 7: Figure S5. Expanded regional plot of Figure S2 showing scenario of admixture for pig breeds and populations in Europe and Russia. [file 12711_2017_345_MOESM7_ESM.tif]

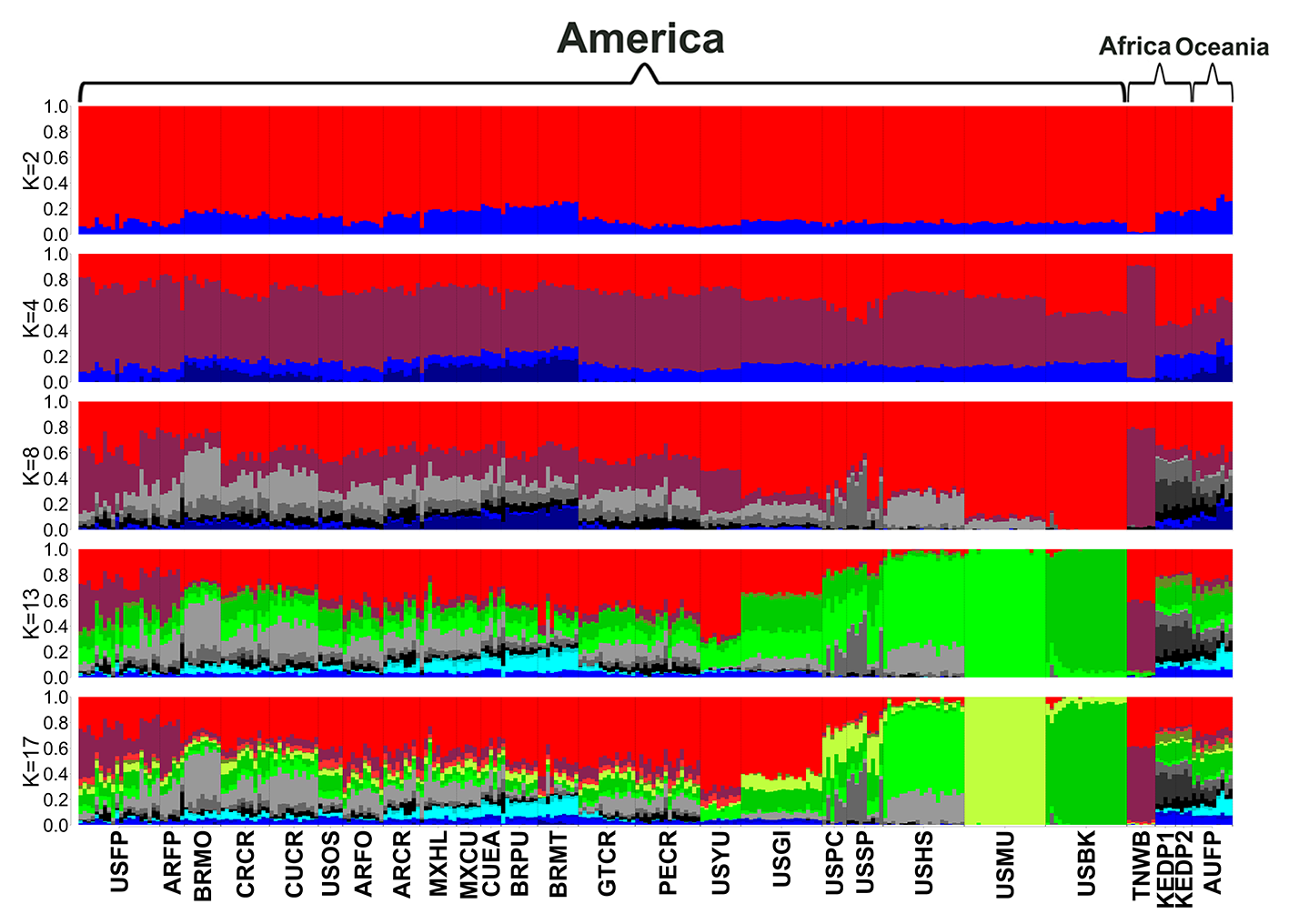

Supplement: Supplementary file 8 — Additional file 8: Figure S6. Expanded regional plot of Figure S2 showing scenario of admixture for pig breeds and populations in North and South America. [file 12711_2017_345_MOESM8_ESM.tif]

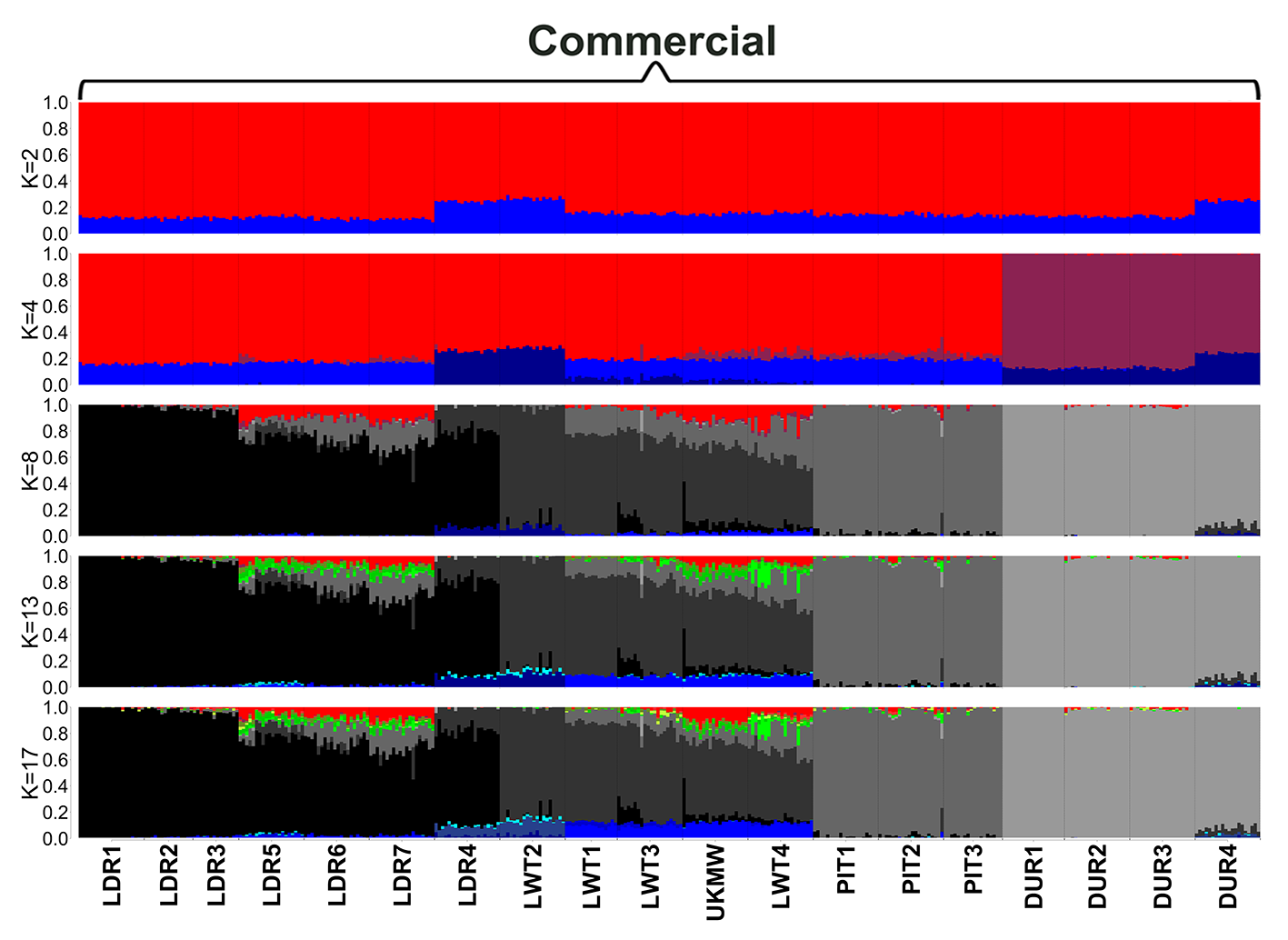

Supplement: Supplementary file 9 — Additional file 9: Figure S7. Expanded regional plot of Figure S2 showing scenario of admixture for pig breeds and populations in African and Oceanian countries. [file 12711_2017_345_MOESM9_ESM.tif]

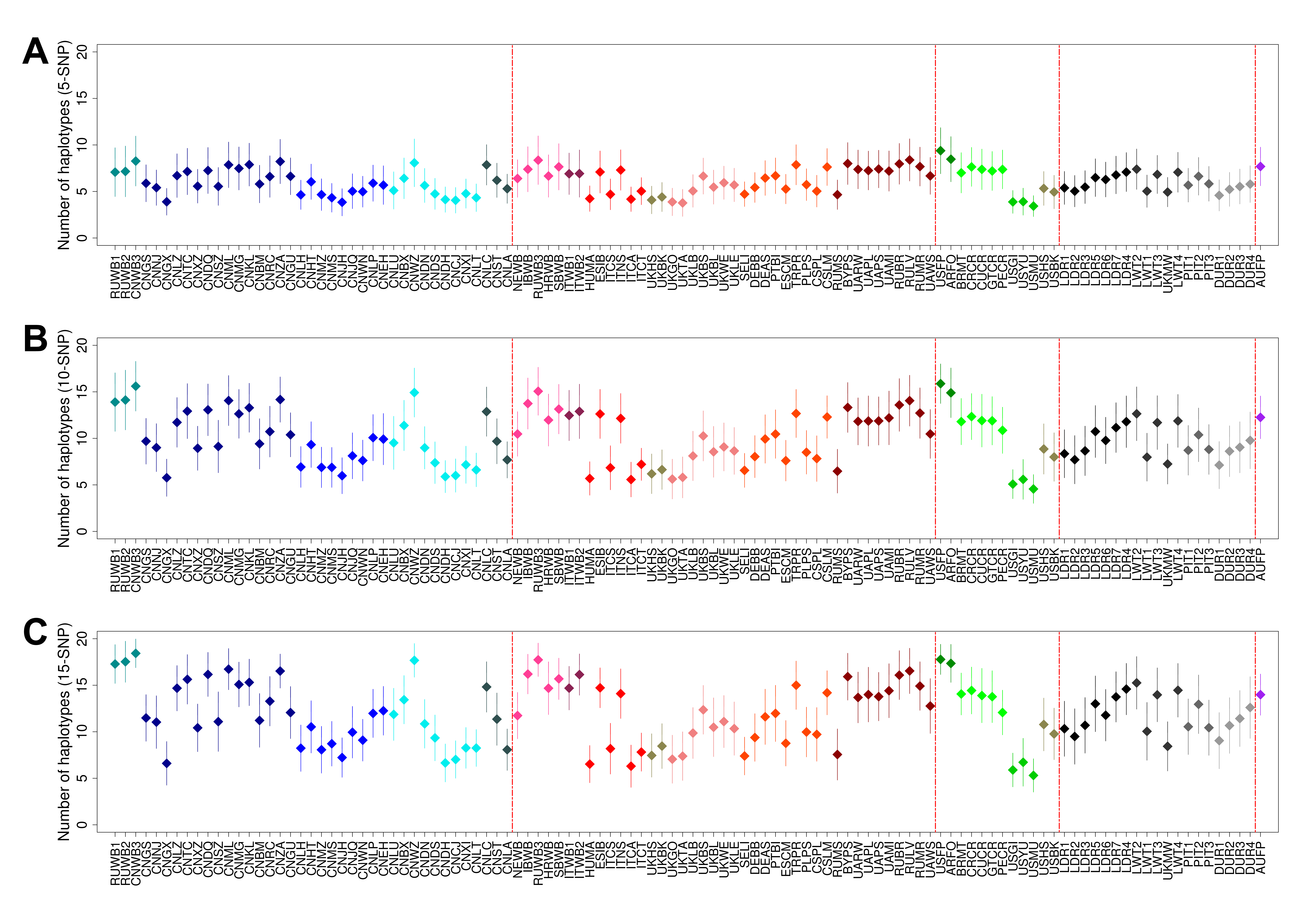

Supplement: Supplementary file 11 — Additional file 11: Figure S9. Distribution of haplotype diversity for pig populations across the world. Diamonds and vertical bars represent means and standard deviations of number of haplotypes respectively in 5-SNP s (A), 10-SNP (B), and 15-SNP (C) windows across the genome for each population with a minimum of 10 individuals. [file 12711_2017_345_MOESM11_ESM.tif]

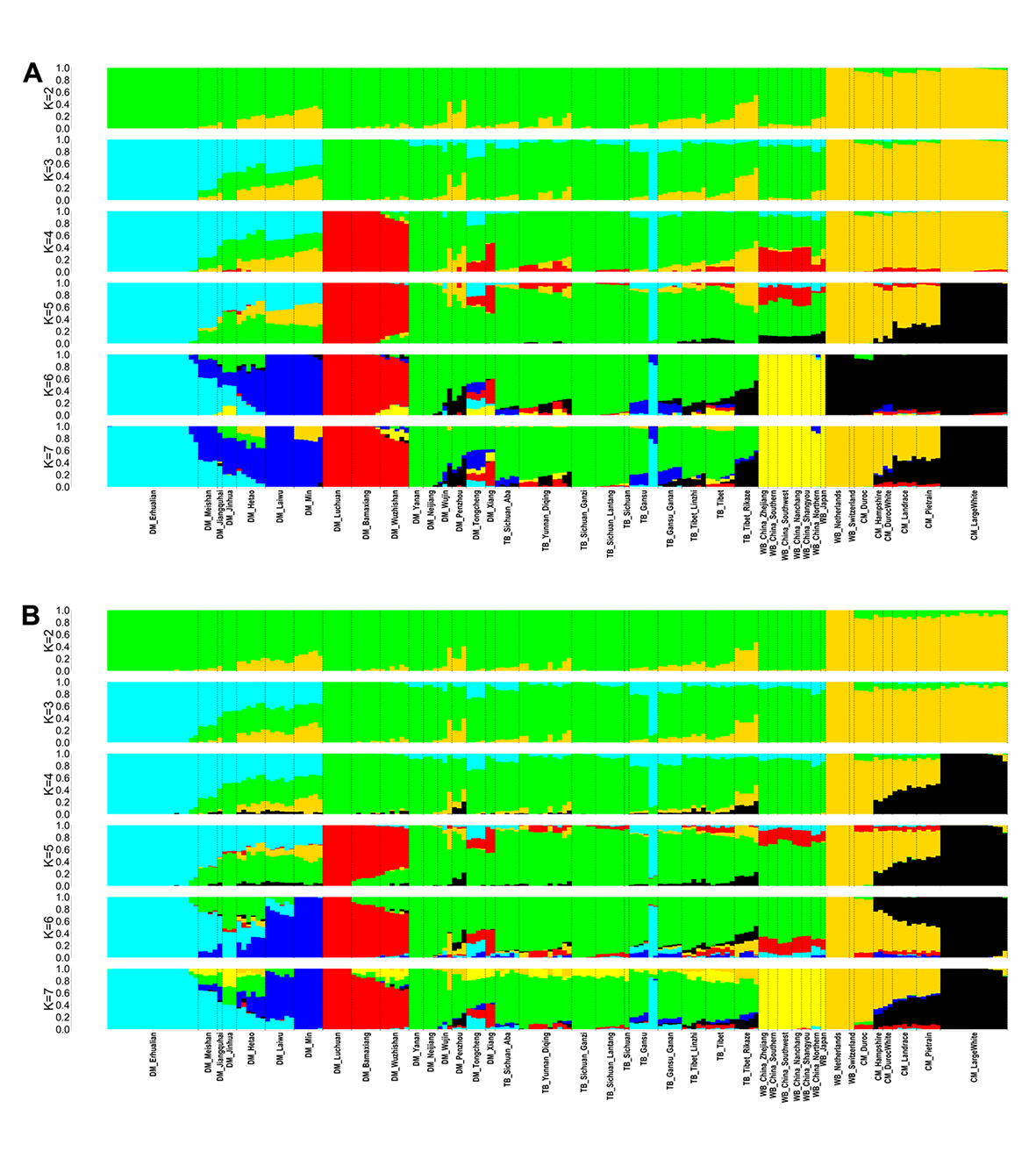

Supplement: Supplementary file 14 — Additional file 14: Figure S12. Results of admixture analysis using 30.4 million SNPs called from whole-genome sequence data (A) were similar to those results obtained using 60K SNP data (B). Whole-genome sequence raw data from 188 individuals mainly obtained from [4, 18, 70], were used in the analysis. The whole-genome SNPs were called using GATK best practice workflow (www.broadinstitute.org/gatk). A total of 30.4 million SNP with a MAF >0.02 and a call rate >70% were kept for admixture analysis (A). A total of 44,988 SNPs with genome positions that were concordant with those of the Illumina 60K SNPs were extracted from the 30.4 million SNP data to represent the results of 60K SNPs (B). [file 12711_2017_345_MOESM14_ESM.tif]

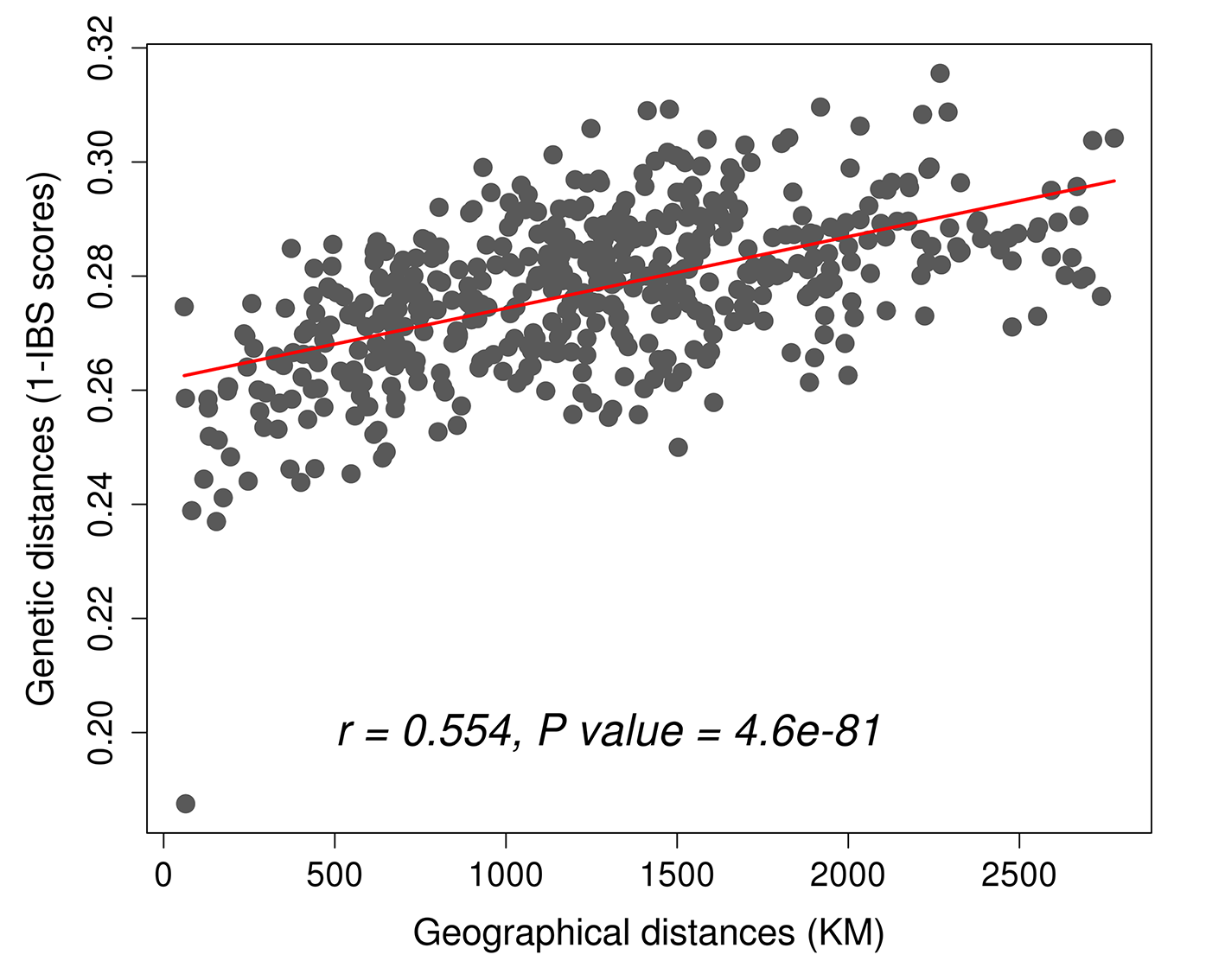

Supplement: Supplementary file 15 — Additional file 15: Figure S13. Scatter plot of geographical distances among pig breeds in China against their genetic distances after removing pigs breeds with more than 20% introgression from European ancestry as revealed by admixture analysis (K = 2). [file 12711_2017_345_MOESM15_ESM.tif]
